# Early arc crust formation preserved in the Grenadines archipelago, southern Lesser Antilles arc

**DOI:** 10.1098/rsos.231914

**Published:** 2024-05-29

**Authors:** Michal Camejo-Harry, Elena Melekhova, Sarah Aufrère, Anders McCarthy, Jon Blundy

**Affiliations:** ^1^ Department of Earth Sciences, University of Oxford, Oxford, OX1 3AN, UK; ^2^ Department of Earth Sciences, Simon Fraser University, , British Columbia V5A 1S6, Canada; ^3^ Department of Earth Sciences, ETH Zurich, Zurich, Switzerland

**Keywords:** volcanic island arc, xenoliths, crustal structure, Lesser Antilles, Grenadines, subduction zone processes

## Abstract

Intra-arc diversity in volcanic activity and composition is ubiquitous, but its underlying causes remain largely unresolved in many settings. In this work, we examine such variability in the Grenadines archipelago, southern Lesser Antilles arc. Here, juxtaposed volcanic centres exhibit eruptive longevities and chemistries distinct from northern counterparts in the same arc. Our goal is to explain this deviation by investigating variations in magmatic processes using petrological data from erupted crustal xenoliths and lavas, and interpreting these findings within the context of the archipelago’s tectonic history and geophysical structure. Textural analyses of xenoliths reveal crystallization over a wide range of pressure–temperature–melt composition conditions in the crust. Mineral phases display discrete compositional trends pointing towards significant inter-island variability in underlying plumbing systems. The geochemical variety of erupted magmas is reminiscent of the entire arc. We speculate that the Grenadines represents the early onset of subduction forming the modern-day Lesser Antilles arc. Extrusive volcanism initiated as submarine activity. Subsequent uplift eroded the original topography of these volcanic centres following the eventual cessation of volcanism in the Neogene. The positioning of the Grenadines on an elevated platform provides rare modern insight into early arc crust formation not commonly preserved in established active arcs.

## 1. Introduction

### 1.1. Overview

Spatio-temporal variations along arc strike are a global phenomenon. Within the same volcanic arc, differences can be observed in relative edifice positioning and size, erupted composition and volume and eruption style. Such variability complicates risk mitigation strategies for communities living near active arc volcanoes given the variety of hazards associated with impending eruptions of an indeterminate nature [1,2]. The driving forces behind intra-arc diversity are largely attributed to variations in crustal properties and mantle flux [3,4]. Deciphering the relative importance of contributing factors by geography is best resolved by integrating datasets of detailed studies on individual volcanoes with regional arc-scale findings of a multi-disciplinary nature.

**Figure 1.**
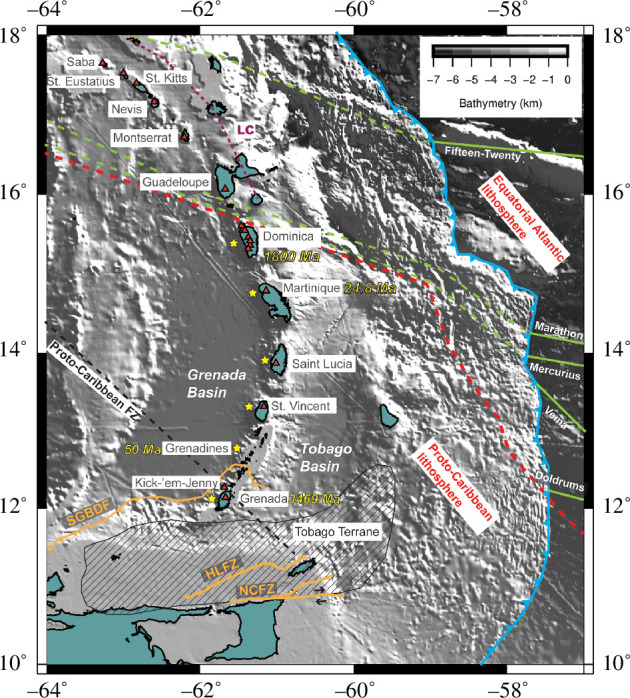
Topographic map of the Lesser Antilles arc generated using Generic Mapping Tools (GMT) [7]. Red triangles denote active volcanoes. Limestone Caribbees are shown by the purple stippled line. The blue line shows the location of the trench. Green solid lines indicate oceanic fracture zones and continuing dashed lines show inferred projection of subducted fracture zones [8]. The black dashed line shows the subducted proto-Caribbean fracture zone [9]. The dashed red line shows the boundary between the proto-Caribbean and equatorial Atlantic seafloor [10]. The hashed region outlines the Tobago Terrane [11]. Orange line shows fault zones mentioned in the text: Southern Grenada Basin deformation front [11], Hinge Line fault zone and North Coast fault zone [12]. Yellow stars highlight islands with reported meta-igneous cumulate xenoliths [13–17]. Yellow text represents ages of the oldest pre-Neogene volcanic rocks identified in the active arc (Grenada, Rojas-Agramonte, Williams [18]; Mayreau, Grenadines [19]; Martinique, Germa, Quidelleur [20]; and Dominica, Frey, Manon [21]).

The Lesser Antilles is a prime example of an active intra-oceanic arc with notable large-scale variations in magmatism and a wealth of available data. Recent geophysical, geochemical and petrological investigations (e.g. VoiLA project [5]) have enhanced scientific understanding of its subduction history, crustal structure, mantle productivity and resultant ranges in eruptive dynamics. The present-day 750 km arc spans islands Saba in the north to Grenada in the south, and represents the culmination of a multi-directional arc front embodying the slow (2 cm yr^−1^ [6]) convergence of Atlantic and proto-Caribbean oceanic lithosphere with the Caribbean plate (figure 1) [10,22]. The overriding latter plate spans 24–37 km in crustal thickness along arc strike [23], with an overall thickening northwards due to the presence of features representative of older episodes of magmatism [22]. There is a north–south variation in the nature of sediment being introduced by the downgoing slab, believed to influence erupted compositions: geochemically, the northern arc has a pelagic marine signature while the southern arc is rich in clastic detritus from the South American continent [24]. The mantle wedge structure is also changeable, with discrete low-velocity anomalies beneath Montserrat, Guadeloupe-Martinique and the Grenadines, attributed to regions of fluid and/or melt accumulation probably enabled by dehydration in nearby fracture zones on the downgoing slab (figure 1) [25,26].

Along-arc variations are controlled by this broad structural framework. In most subduction zone settings, surface volcanism originates through the injection of hydrous, mantle-derived basalts into a deep crustal hot zone [27]. Higher quantities of slab-derived water lower the melting point of mantle rocks promoting melt generation. In the Lesser Antilles, there is a demonstrable correlation between magma production rates and the delivery of water to the mantle wedge, facilitated by serpentine dehydration in subducted fracture zones [9]. Subsequent magmatic differentiation to produce more evolved magma takes place within the arc crust. In the Lesser Antilles, a four-layer structure, related to magmatic differentiation processes, has been proposed: (i) uppermost layer (5 km thick) composed of loosely consolidated and fractured volcaniclastics and sediments; (ii) upper (6–15 km); (iii) middle (12–32 km); and (iv) lower (26–38 km) layers composed of plutonic igneous rocks with considerable inter-island compositional variation [28]. Variability in the compositions and depths of crustal igneous layers (ii)–(iv) reflect differences in magmagenesis and differentiation beneath each volcanic island [23,28].

A wide range of magma types is observed across the Lesser Antilles arc: tholeiitic suites dominate the northern islands (Saba to Montserrat), calc-alkaline suites characterize the central islands (Guadeloupe to Saint Lucia) and silica-undersaturated varieties occur in the southern islands (St Vincent to Grenada) [29]. In terms of rock types, erupted andesites are predominant in northern and central islands while basalts and basaltic andesites predominate in southern islands [30]. These compositional variations are mainly a result of polybaric differentiation within the crust [31]. The extent to which chemical diversity is further influenced from subducted sediments sources in the mantle wedge or crustal contamination has long been debated [30,32,33]. Yet, despite subducted sediment signatures varying north to south [30,34], assimilation of pre-existing crustal rocks has been shown to hold higher significance [35], with lateral variations in crustal structure along arc strike emphasizing how wide-ranging differentiation mechanisms can be [28].

**Figure 2.**
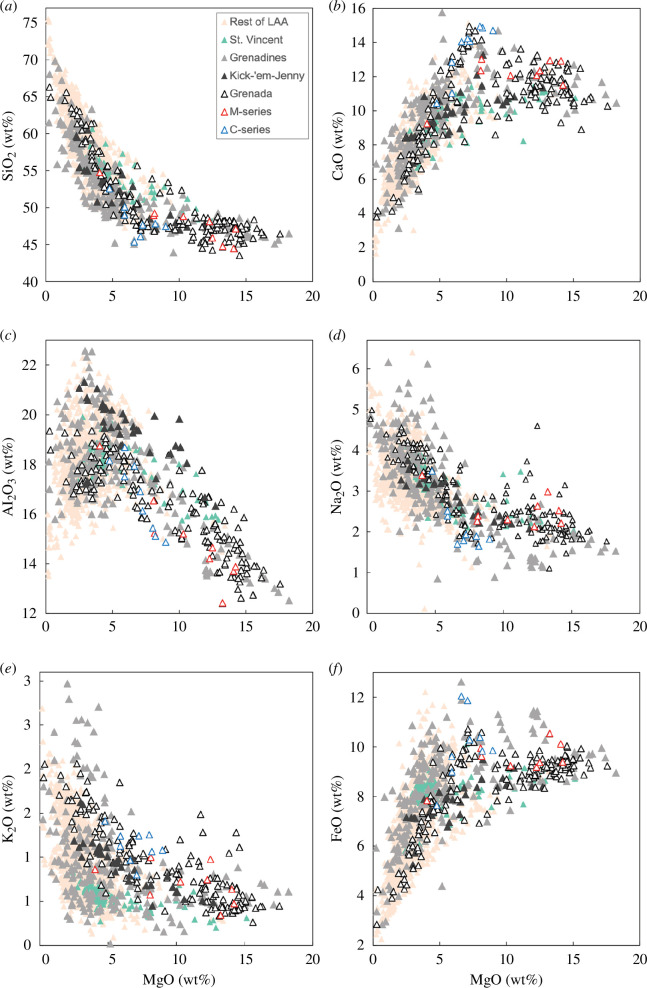
Bulk-rock major element variations in Lesser Antilles lavas. Data are taken from Aufrère [36], Camejo-Harry *et al.* [15], Camejo-Harry *et al.* [37], Devine and Sigurdsson [38], Sigurdsson and Shepherd [39], T. Smith (2020) unpublished, Westercamp *et al.* [40], White *et al.* [13] and the GEOROC database. Grenadines lavas are from All Awash Island, Bequia, Canouan, Carriacou, Diamond Island, Frigate Island, Île de Caille, Île de Ronde, Les Tantes, Mayreau, Mustique, Petit Canouan, Petite Martinique, Petit Nevis, Petit St Vincent, Saline Island, Savan and Union. M- and C-series basalts from Grenada are distinguished using data from White *et al.* [13].

Southern Lesser Antilles volcanic centres exhibit a number of intriguing features. As highlighted above, constituent volcanoes have geochemical signatures that are distinct from their northern counterparts erupting predominantly basalts and basaltic-andesites, including primitive, hydrous and MgO-rich (greater than 12 wt%) basalts (figure 2) [30]. The mineralogy of crustal xenoliths differs considerably in being more mafic (plagioclase-poor) to the south, contrasting with central and northern segments where felsic phases (notably plagioclase) predominate [28]. There are also substantial differences in surface activity. Historically (seventeenth century onwards), there have been several volcanic eruptions and volcano-seismic crises on the majority of islands making up the active arc [41,42]. However, islands of the Grenadines archipelago which are among the smallest in size (figure 1), are exceptions to this overall behaviour. Subaerial Grenadines volcanism is largely restricted to the Neogene period and detected seismicity has been minimal. At the southern terminus of the arc segment, on the island of Grenada, volcanism has occurred over the past 6 Ma [13], but historical activity has only been manifested as hot springs and periodic earthquake swarms [43]. This contrasts with the frequently active La Soufrière volcano on the island of St Vincent erupting in 1718, 1812, 1902/1903, 1971/1972, 1979 and 2020/2021, and submarine Kick-’em-Jenny volcano, considered to be a part of the Grenadines [44], erupting 14 times since its discovery in 1939. The geographical ‘anomaly’ that is the inactive subaerial Grenadines is a key motivator for this study.

**Figure 3.**
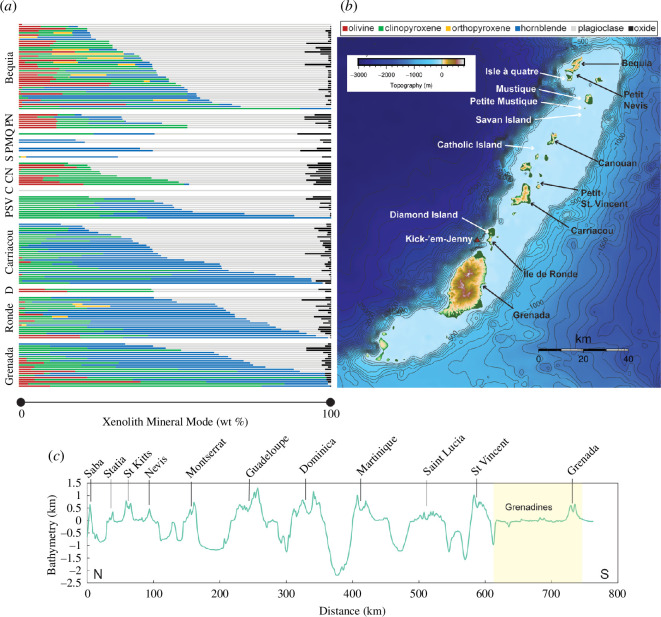
(*a*) Modal proportions (by mass) of xenolith mineral phases from representative islands in Grenada, the Grenadines and St Vincent. By locality, samples ranked in order of increasing proportion of mafic minerals down the stacked bar chart. C, Catholic Island; CN, Canouan; D, Diamond Island; M, Mustique; P, Petite Mustique; PN, Petit Nevis; PSV, Petit St Vincent; Q, Isle à Quatre; S, Savan Island. Data are taken from this study, Stamper *et al.* [45], Camejo-Harry *et al.* [15], Melekhova *et al.* [28], Aufrère [36], Camejo-Harry [46], Melekhova *et al.* [14] and unpublished data from T. Smith (2020). Note that overall, their mafic nature increases from north to south decreasing plagioclase proportions. (*b*) Topographic map (generated using GMT, Wessel *et al.* [7]) highlighting locations of these islands, together with Kick-’em-Jenny submarine volcano (red triangle). Contours are in metres. (*c*) Along-arc bathymetry constructed using GeoMapApp with latitude-longitude elevation data from the global multi-resolution topography (GMRT). Grenada and the Grenadines are highlighted in yellow.

Upon this backdrop, we investigate variations in magmatic processes in the Grenadines archipelago using petrological data from erupted crustal xenoliths. We compile new textural and compositional xenolith data from previously understudied Grenadines islands and integrate them with published data from neighbouring islands within the archipelago, including Grenada. We then integrate these findings with published geochemical data from lavas and interpret within the context of the tectonic history and geophysical structure of the southern Lesser Antilles. We aim to answer two questions: (i) Are the subaerial Grenadines fed by the same magma crustal plumbing system? (ii) Does the bathymetric delineation of Grenada and the Grenadines (figure 3*b*) translate to a shared magma plumbing system?

### 1.2. Regional setting and background

The Lesser Antilles arc has had a wide-ranging tectonic history stemming from its Pacific origin as part of the Great Arc of the Caribbean during the Cretaceous, and subsequent eastward migration into the Atlantic on the leading edge of the Caribbean Plate as it emplaced between North and South America during the Eocene [47,48]. Proto-Caribbean lithosphere previously occupying the Atlantic seaway has mostly subducted beneath the Caribbean Plate, with lithosphere formed originally at the Mid-Atlantic ridge now entering the trench along most of the Lesser Antilles arc [10]. Remaining fragments of proto-Caribbean crust (Mesozoic) escaping subduction are suggested to have either accreted to the Caribbean Plate on the present-day Lesser Antilles or thrust onto northern South America [49].

The Lesser Antilles arc’s Eocene history reveals a tectonic duality in its back-arc region: a compressional regime in the north versus extension in the south [50]. The Grenada Basin, located west of the present-day southern Lesser Antilles (figure 1), is floored by oceanic crust, and considered to be a manifestation of this extensional regime, originally extending beneath the Grenada–Martinique region before the development of the current arc [51–53]. Many models for the formation of the Grenada Basin centre around rifting and seafloor spreading, with details varying in the direction of opening relative to the active arc, i.e. fore-arc [54] versus back-arc [22] extension.

For the past 60 Ma, the Caribbean Plate has also interacted with the northern boundary of the South American Plate with numerous mapped fault systems taking up strike-slip motion [52,55]. The southeast corner of this plate boundary zone is occupied by the Tobago Terrane (figure 1). This is an extensive lithospheric block (bounded to the south by the North Coast fault zone) consisting of an oceanic basement (Cretaceous or earlier) overlain by material derived from widespread island arc magmatism and marine sedimentation [11]. The terrane’s northern extent has been debated, with some factions proposing based on geophysical data the Hinge Line fault zone [12,52] and others suggesting a northwest progression beneath the southern Lesser Antilles either as an isolated fragment within accretionary fore-arc or exposed Mesozoic igneous and metamorphic basement terrane [19,56]. North of the Tobago Terrane, the plate boundary zone is delineated by the southern Grenada Basin deformation belt interpreted as an accretionary prism caused by the subduction of the Grenada Basin lithosphere beneath the northern margin of the Tobago Terrane [11].

The Lesser Antilles arc platform is a ridge separating fore-arc and back-arc oceanic basins upon which today’s modern arc is built (figure 1) [19]. Its southernmost extent is bathymetrically defined (figure 3*b*), thought to have uplifted before the extrusion of volcanic islands Grenada and the Grenadines. Prior to the onset of Miocene volcanism, the southern Lesser Antilles arc platform (SLAAP) uplifted in an east-tilted half horst during the late Oligocene to early Miocene boundary [19]. The geology of Grenada and the Grenadines therefore records a mixture of diverse igneous and sedimentary rocks denoting changes in magmatism and depositional environments over time, facilitated by regional deformation. Four key volcanic influences have been identified in the region’s rock record: (i) middle Eocene back-arc spreading centre in the Grenada Basin (oldest exposure is the Mayreau (pillow) Basalt erupted 50–46 Ma); (ii) Palaeogene magmatic arc in the Grenada Basin depositing copious volcanogenic sediments; (iii) in-place upper Oligocene igneous intrusions of unknown tectonic origin (e.g. 38 Ma dacitic dike cutting the sedimentary Tufton Hall formation on Grenada); and (iv) Miocene (onset 12–15 Ma) arc magmatism resulting in the extrusion of the modern Lesser Antilles [13,19,22,57,58]. Some Grenadines islands (e.g. Battowia, Baliceaux, Mustique, Savan and Tobago Cays) did not experience post-Oligocene volcanism [19,40,56,59]. For the Grenadines, Neogene volcanism was mainly intrusive (dikes, sills, plutons and diatremes), only transiting to the surface less than 9 Ma [19]. Conversely for Grenada, Neogene and Quaternary extrusions cover most of the island [60].

Available geochronological data suggests that volcanic activity emanating from the Miocene in Grenada and the Grenadines was contemporaneous yet unsystematic. Grenada volcanism took place across five centres beginning at 6 Ma and continuing into the Quaternary [13,60]. Immediately north, lavas from Île de Ronde date from 3.2–4.6 Ma [40] with no geological evidence for Quaternary volcanism. Meanwhile, neighbours Île de Caille and Kick-’em-Jenny are markedly younger, having eruptions as recently as approximately 1000 AD [61] and 2017 [62], respectively. Further northwards, radiometric ages of volcanic rocks include 2.7–11.2 Ma for Carriacou [19,57], 6.8 Ma for Canouan [40] and 3.5–5 Ma for Bequia [57]. It should be noted that many of the remaining Grenadines have never been subjected to radiometric determination.

### 1.3. A stand-alone southern Lesser Antilles?

The southern Lesser Antilles has consistently been shown to present different characteristics from its northern counterparts; however, the geographical boundary for these transitions relies heavily on the parameter being investigated. For example, Saint Lucia to Grenada have been grouped based on matching subduction angles and magma production rates [63,64], whereas St Vincent to Grenada have been bracketed based on the predominance of mafic rock types (figure 2) [65]. Attempts at geochemical groupings for erupted material have been less straightforward [30]. Brown *et al.* [29] speak to a chemical variation along the arc axis with St Vincent being transitional between southern and central affinities consistent with its geographical location. However, these broad generalizations mask inter-island variations, such as the local significance M- and C-series magmas hold to Grenada. These two distinct magma series have erupted contemporaneously from Grenada’s five volcanic centres over the past 6 million years: (i) picritic M-series with olivine microphenocrysts and (ii) ankaramatic C-series with large clinopyroxene phenocrysts, enriched in calcium and strontium [60,66,67]. Highly magnesian M-series basalts include the most primitive lavas of the Lesser Antilles.

Past tectonics also plays a role in grouping islands of the southern Lesser Antilles. The wider Grenada to Dominica region can be banded together based on some key characteristics. First, this section of the arc occupies the axis of previous back-arc spreading [22]. It is therefore not a coincidence that the oldest volcanic material (pre-Miocene), including xenocrystic zircons (Precambrian to Eocene) in the Grenadines [18] and Dominica [21], has only been observed in this part of the arc (ages displayed in figure 1). This is also the domain through which Proto-Caribbean lithosphere is subducted beneath the arc (figure 1). This matches identified incidences of meta-igneous cumulate xenoliths (yellow stars in figure 1), a feature which has been ascribed to older arc crust (Proto-Caribbean) present at the onset of subduction [14].

Specifying a southern segment with exclusive characteristics is therefore not clear cut due to the range of competing interactions influencing patterns along arc strike. To simplify matters, and for the purpose of this study, we take a broad view in distinguishing the southern Lesser Antilles as Grenada and the Grenadines based on geographic proximity, compositional similarities and bathymetry.

### 1.4. Magma storage systems

It is now widely accepted that the magmatic systems feeding volcanoes are vertically extensive, rooted at the crust–mantle boundary and straddling the entire crust [68]. These systems are dominated volumetrically by deformable crystalline mushes composed of interlocking networks of crystals through which buoyant melts and fluids of varying compositions and proportions are distributed. The accumulation of eruptible melt-rich domains within magma reservoirs is facilitated by incremental magma recharge [69], remobilization of cold crystal-rich mushes by reheating [70,71] and/or reactive melt flow through the mush [72]. Without sufficient heat supply, the majority of a magma system’s lifetime can be spent in a highly crystalline state with eruptible magma bodies or lenses developing locally and ephemerally [73,74].

Within the Lesser Antilles arc, eruptive patterns have been reconciled with the presence of long-lived trans-crustal mush systems [42]. Metcalfe *et al.* [42] distinguish three broad levels of magma storage across the arc: (i) greater than 18 km, (ii) 10–18 km and (iii) less than 10 km, with inter-island variability in their relative depths. The information required to develop these models can be obtained from monitoring data [75], phase equilibria experiments [76,77] and chemistry of erupted material [37,78,79]. Crustal xenoliths and lavas are worthy specimens of the latter. Xenoliths sample the crystalline roots of magmatic systems and include cumulate residues from crystal fractionation and plutonic equivalents of erupted magma, while lavas as differentiated end-products derive from eruptible magma storage reservoirs. These distinct yet complementary rock archives can help unravel the complexity of processes operating within underlying trans-crustal magmatic systems [15,45]. As the Lesser Antilles is renowned for its unusual abundance of crustal xenoliths and chemical diversity of lavas [16,30], it offers a unique opportunity to unravel the intricate processes operating within underlying trans-crustal magmatic systems.

## 2. Methods

### 2.1. Data compilation

Whole rock lava data from the current Lesser Antilles arc are taken from Aufrère [36], Camejo-Harry *et al.* [15, 37], Devine and Sigurdsson [38], Sigurdsson and Shepherd [39], Westercamp *et al.* [40], White *et al.* [13] and the GEOROC database (https://georoc.eu/) in 2020 using the following parameter: Geological setting = Convergent margins − Lesser Antilles. Data from Aufrère [36] and T. Smith unpublished (2020) are presented in electronic supplementary material, table S1. Existing crustal xenolith textural and compositional data are compiled for several volcanic centres in the southern Lesser Antilles: Bequia [15], Canouan [36], Petit St Vincent [14], Carriacou [28], Île de Ronde [46] and Grenada [45]. Unpublished data for Bequia and Petit Nevis are provided by T. Smith (2020) and are included in electronic supplementary material, table S2.

### 2.2. Analytical techniques

New data were obtained for crustal xenoliths from Grenadines islands Isle à Quatre, Mustique, Petite Mustique, Savan Island, Catholic Island, Carriacou and Diamond Island. Analyses were conducted at the Universities of Oxford and Bristol. Petrographic observations were carried out on thin sections of these samples using optical microscopes from which representative xenolith samples from each locality were chosen for analysis based on textures and mineral assemblages. Modal abundances of the major mineral phases in representative xenoliths were obtained by point counting (greater than 500 points per sample). At Oxford, scans of thin sections were point counted using a random grid in *JMicroVision* version 1.3.4 [80]. At Bristol, point counting was done using a mechanical stage. Volume modes were used to classify xenoliths after the scheme of Streckeisen [81] (electronic supplementary material, table S2) and then converted to mass modes using mineral densities (figure 3*a*, electronic supplementary material, table S2) [82].

Polished, carbon-coated thin sections were imaged using scanning electron microscopes FEI Quanta 650 at the University of Oxford and Hitachi S-3500N at the University of Bristol. Major element concentrations of minerals were then analysed on thin sections by electron probe micro-analysis using a Cameca SX-5 FE at Oxford and Cameca SX100 at Bristol. Mineral analytical conditions included 15 kV accelerating voltage, 4 nA beam current and 5 μm spot size at Oxford, 20 kV accelerating voltage, 10 nA beam current and 1 μm spot size at Bristol. Primary calibrations were carried out using Albite (Na, Si), MgO (Mg), TiO_2_ (Ti), Sanidine (K), Wollastonite (Ca), Fayalite (Fe) and elemental Mn, Ni and Cr at Oxford and Amelia Albite (Na, Si), St John’s Olivine (Mg), Eifel Sanidine (Al, K), Wollastonite (Ca), Ilmenite (Ti, Fe), Cr_2_O_3_ (Cr) and elemental Mn and Ni at Bristol. Peak counting times for mineral analyses at Oxford were 20 s for Na, 45 s for Mg, 40 s for Si, Al and Ti and 30 s for K, Ca, Mn, Fe, Ni and Cr. At Bristol, peak counting times were 10 s for Na, Mg, Al, K, Ca, Ti, Cr, Fe, Mn and Ni. Ferric iron contents were estimated using the stoichiometric methods of Droop [83] for spinel, Lindsley [84] for clinopyroxene and Holland & Blundy [85] for hornblende.

## 3. Results

To assess along-arc variations in magmatism within the southern Lesser Antilles, we first present petrographical and compositional xenolith data from Grenadines islands Isle à Quatre, Mustique, Petite Mustique, Savan Island, Canouan, Catholic Island, Carriacou, Diamond Island and Île de Ronde. Next, we synthesize all available petrological data from the remaining islands and report below.

Textures and bulk compositions were used subdivide xenoliths into three groups: (i) *igneous cumulates*, xenoliths having cumulate textures and representing instantaneous solid extracts from magma; (ii) *hypabyssal*, with textures consistent with shallow emplacement and rapid crystallization of melts; and (iii) *meta-igneous cumulates*, cumulate xenoliths displaying evidence of recrystallization and deformation (after Melekhova *et al.* [14]). These designations are used throughout the paper. While identifying meta-igneous cumulate xenoliths was done via textural observation, distinguishing between igneous cumulate and hypabyssal xenoliths was confirmed using bulk compositions. Bulk rock major element compositions of xenoliths were calculated using averaged mineral compositions and mass fraction modes. Where olivine compositions could not be accurately measured due to alteration, calculations were not attempted. Igneous cumulate xenoliths are discriminated as those having bulk compositions on a tangent to the liquid line of descent of lavas. In contrast, hypabyssal xenoliths though texturally plutonic, lie along the liquid line of descent of lavas.

### 3.1. Petrography

Across the nine studied islands, xenoliths contain spinel, olivine (partially to completely iddingsitized), plagioclase, clinopyroxene and hornblende. Minor exceptions occur with the presence of ilmenite in Canouan and Mustique, sulphides in Carriacou and orthopyroxene in Île de Ronde and Savan Island. Accessory phases such as quartz are present in Mustique, Carriacou and Diamond Island, titanite in Mustique and apatite and biotite in Carriacou and Île de Ronde. Some localities show additional alteration minerals (e.g. Mustique, Petite Mustique, Canouan and Carriacou) with occurrences of zeolites, carbonates, chlorite and/or sericite. Igneous cumulate xenolith rock types include (i) gabbros (with minor gabbronorites, troctolites and anorthosites) for Isle à Quatre, Mustique, Petite Mustique, Savan Island, Canouan, Catholic Island and Diamond Island; (ii) hornblendites, clinopyroxenites and gabbros for Carriacou; and (iii) hornblendites, gabbros and gabbronorites for Île de Ronde (electronic supplementary material, table S3). This geographical gradation is mirrored in terms of mineral modes, with an overall north–south decrease in the dominance of plagioclase and an increase in clinopyroxene/hornblende proportions (figure 3*a*).

The relative crystallization order of xenolith phases, determined from textural observations of included and interstitial phases, is variable (electronic supplementary material, table S3), so too are the phase assemblages present within individual xenoliths (table 1). This is further complicated by instances of multiple generations of a phase within the crystallization sequence (electronic supplementary material, table S3). Zoning and melt inclusions are common (the latter are not described in detail here).

**Table 1.** Summary of mineral assemblages and compositions in Grenadines xenoliths.

locality	suite	assemblage	olivine	plagioclase	clinopyroxene	hornblende	spinel
Isle à Quatre	igneous cumulate	Ol + pl + cpx + hbl + spl	n.d.	An 90–94	Mg# 73–80	Mg# 67–70	Cr# <8
		Ol + pl + cpx + hbl	n.d.	An 94–96	Mg# 77–84	Mg# 71–74	n.d.
Mustique	igneous cumulate	Ol + pl + cpx + spl + qz	n.d.	An 91–93	Mg# 79–81	n.d.	n.d.
	hypabyssal	Pl + hbl + spl + ilm + qz± ttn	n.d.	An 43–54	n.d.	Mg# 63–82	Cr# <30
Petite Mustique	igneous cumulate	Pl + hbl + spl ± cpx	n.d.	An 89–92	n.d.	Mg# 63–68	Cr# <1
		Pl + hbl + spl	n.d.	An 88–93	n.d.	Mg# 67	Cr# <1
Savan Island	igneous cumulate	Pl + opx + hbl + spl ± cpx ± ol	n.d.	An 90–91	Mg# 77	Mg# 69–70	Cr# <1
Canouan	igneous cumulate	Pl + cpx + spl	n.d.	An 63–96	Mg# 66–78	n.d.	Cr# <7
		Ol + pl+ cpx + spl	Fo 74–77 NiO_2_ <0.04 wt%	An 68–94	Mg# 71–79	n.d.	Cr# <8
		Ol + pl + cpx + hbl + spl	Fo 74–76 NiO_2_ <0.03 wt%	An 87–93	Mg# 76–79	Mg# 70	Cr# <1
	hypabyssal	Pl + cpx + spl	n.d.	An 40–89	Mg# 69–78	n.d.	Cr# <3
Catholic Island	igneous cumulate	Pl ± ol	n.d.	An 95–97	n.d.	n.d.	n.d.
Carriacou	igneous cumulate	Pl + cpx + spl	n.d.	An 13–90	Mg# 70–88	n.d.	Cr# <33
		Pl + cpx + hbl + spl	n.d.	An 36–96	Mg# 61–90	Mg# 31–74	Cr# <43
		Pl + cpx + hbl + spl + qtz	n.d.	An 21–93	Mg# 73–81	Mg# 59–71	n.d.
		Pl + cpx + hbl	n.d.	An 61–95	Mg# 78–81	Mg# 68–76	d.
		Pl + hbl + spl	n.d.	n.d.	n.d.	Mg# 62–66	n.d.
		Pl + hbl + spl + qtz	n.d.	An 19–62	n.d.	Mg# 60–71	n.d.
		Ol + pl + cpx + hbl + spl	n.d.	n.d.	n.d.	Mg# 70–71	Cr# <4
	hypabyssal	Ol + pl + cpx + spl	Fo 70–91 NiO_2_ <0.35 wt%	An 37–90	Mg# 71–86	n.d.	n.d.
	meta-igneous cumulate	Pl + cpx + hbl + spl	n.d.	An 51–96	Mg# 62–84	Mg# 49–77	Cr# <27
		Pl + cpx + hbl	n.d.	An 92–94	Mg# 76–80	Mg# 67–72	n.d.
		Pl + hbl + spl	n.d.	An 90	n.d.	Mg# 67–74	n.d.
		Cpx + hbl + spl	n.d.	n.d.	Mg# 75–80	Mg# 66–70	Cr# <30
Diamond Island	igneous cumulate	Ol + pl + cpx + spl	Fo 76–86 NiO_2_ <0.13 wt%	An 65–88	Mg# 71–77	Mg# 70–74	Cr# <9
	hypabyssal	Pl + cpx + hbl + spl ± ol ± qz	n.d.	An 37–73	Mg# 63–78	n.d.	Cr# <18
Île de Ronde	igneous cumulate	Pl + cpx + hbl + spl ± opx	n.d.	An 29–92	Mg# 76–86	Mg# 60–84	Cr# <80
		Ol + pl + cpx + hbl + spl	n.d.	An 45–94	Mg# 72–86	Mg# 60–80	Cr# <70
		Ol + pl + cpx + opx + hbl + spl	n.d.	An 63–92	Mg# 81–82	Mg# 67–73	Cr# <57
		Ol + pl + hbl	n.d.	An 49–75	n.d.	Mg# 75–79	n.d.
		Pl + cpx + hbl + spl	n.d.	An 55–94	Mg# 71–85	Mg# 59–75	Cr# <66

*Notes:* n.d., not determined.

#### 3.1.1. Isle à Quatre

**Figure 4.**
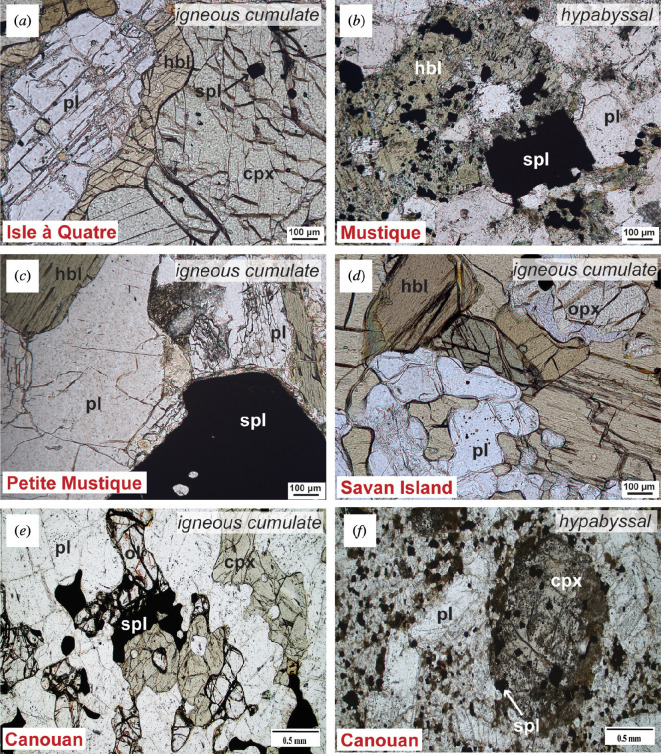
Representative photomicrographs of crustal xenolith textures in plane-polarized light. (*a*) Isle à Quatre olivine-bearing pyroxene hornblende gabbro, igneous cumulate (IQ2). (*b*) Mustique hypabyssal leuco hornblende gabbro (MST5). (*c*) Petite Mustique hornblende gabbro, igneous cumulate (PMST1.1). Note plagioclase inclusions in spinel. (*d*) Savan leuco hornblende gabbronorite, igneous cumulate (SA1). (*e*) Canouan olivine gabbro, igneous cumulate (CN10). Olivine is partially iddingsitized. (*f*) Canouan hypabyssal gabbro (CN5).

Isle à Quatre xenoliths are of the igneous cumulate variety comprising pyroxene hornblende gabbros (± olivine) and hornblende gabbros (figure 4*a*, electronic supplementary material, table S3a). Textures are mesocumulate and orthocumulate. Oxides (less than 1 mm), when present, are subhedral/anhedral occurring either as intercumulus phases with inclusions of olivine and clinopyroxene or as inclusions (less than 400 μm). Olivine, completely altered to iddingsite, occurs either as anhedral grains (less than 2 mm) with inclusions of other phases (oxides, plagioclase and clinopyroxene) or simply as inclusions (less than 400 μm). Plagioclase grains are subhedral/anhedral occurring either as (i) inclusions (less than 400 μm) or chadacrysts (less than 2 mm) or (ii) as intercumulus phases with sutured contacts (less than 2 mm). Larger-sized grains are generally sieve textured (sometimes heavily resorbed). Clinopyroxene (less than 1.5 mm) grains are subhedral/anhedral, often showing uralitization. Hornblende exists either as prismatic grains (less than 2 mm) with minor inclusions of all other phases or as oikocrysts. Crystallization sequences vary by textural type: the mesocumulate-textured xenolith has a definitive sequence of olivine, clinopyroxene, oxide, hornblende and plagioclase, while orthocumulate-textured xenolith phase sequences are less clear with early co-appearances of oxide, olivine, plagioclase and clinopyroxene (order of crystallization unknown) followed by hornblende and/or second generations of plagioclase and olivine.

#### 3.1.2. Mustique

Mustique xenoliths are igneous cumulate and hypabyssal (figure 4*b*) with mesocumulate textures (electronic supplementary material, table S3*b*). The igneous cumulate xenolith is a leuco olivine gabbro. Olivine is completely altered to iddingsite and anhedral, occurring either as isolated grains (less than 3 mm) or inclusions in all phases. Plagioclase occurs either as anhedral inclusions (less than 600 μm) in clinopyroxene and/or hornblende, or subhedral intercumulus grains with sieve-textured cores (less than 6 mm). Clinopyroxene grains are subhedral/anhedral (less than 6 mm) with inclusions of olivine and plagioclase. The crystallization sequence for this sample is olivine, plagioclase and clinopyroxene, followed by a second generation of plagioclase.

Hypabyssal samples are leuco hornblende gabbros. Oxides (less than 500 μm) are subhedral/anhedral and included in all phases, while also existing as isolated grains. Plagioclase exists as subhedral intercumulus grains with sieve-textured cores (less than 4 mm). Clinopyroxene is mostly replaced by hornblende during uralitization. Hornblende (less than 3 mm) is subhedral/anhedral with inclusions of all other phases. Oxides and plagioclase are the first phases to crystallize, varying in order depending on the sample. This sequence is then followed by hornblende with or without a second generation of plagioclase.

#### 3.1.3. Petite Mustique

Petite Mustique xenoliths are all igneous cumulate hornblende gabbros with mesocumulate textures (figure 4*c*, electronic supplementary material, table S3*c*). Oxides (less than 2 mm) are anhedral and intercumulus with inclusions of plagioclase and clinopyroxene. Plagioclase exists as either subhedral grains with sutured contacts (less than 4 mm) containing minor hornblende inclusions or as anhedral inclusions (less than 200 μm) in hornblende and spinel. Clinopyroxene (less than 200 μm) exists only as minor inclusions in spinel and hornblende. Hornblende (less than 14 mm) is subhedral and prismatic with inclusions of other phases. The major crystallization sequence starts with plagioclase and/or clinopyroxene, when present, followed by the co-appearance of oxides and hornblende, then a second generation of plagioclase.

#### 3.1.4. Savan Island

The lone xenolith studied from Savan Island is a leuco hornblende gabbronorite igneous cumulate with a mesocumulate texture (figure 4*d*, electronic supplementary material, table S3*d*). Oxides (less than 2 mm) are anhedral, existing either as inclusions in all phases or intercumulus. Olivine (less than 200 μm) is minor, anhedral, completely altered to iddingsite and included in hornblende and clinopyroxene. Plagioclase (less than 2 mm) exists as anhedral inclusions in hornblende and subhedral intercumulus phases with sutured contacts and sieve-textured interiors. Both clinopyroxene and orthopyroxene are present, with the former existing mainly as uralitization textures in hornblende and the latter as anhedral inclusions in hornblende, itself having oxide inclusions (less than 2 mm). Hornblende grains are subhedral (less than 4 mm) with poikilitic textures. The crystallization sequence is early co-appearance of oxides and olivine, followed by the co-appearance of plagioclase, clinopyroxene and orthopyroxene, followed by hornblende and a second generation of plagioclase.

#### 3.1.5. Canouan

Canouan xenoliths include igneous cumulate (figure 4*e*) and hypabyssal (figure 4*f*) varieties (electronic supplementary material, table S3*e*). Igneous cumulate xenoliths display mesocumulate to orthocumulate textures, while the lone hypabyssal sample is porphyritic phaneritic.

Igneous cumulate xenoliths are olivine gabbros, gabbros and troctolites, some with leuco designations. Oxides (generally less than 1 mm and up to 4 mm in coarse-grained samples) are subhedral/anhedral and occur either as inclusions in all other phases or as interstitial phases with inclusions of olivine, plagioclase and/or clinopyroxene. Olivine (less than 3 mm) is partially to completely iddingsitized, occurring as either anhedral inclusions in all other phases or subhedral/anhedral intercumulus grains that may have inclusions of plagioclase, clinopyroxene and/or spinel. Plagioclase (generally less than 3.2 mm, but up to 1 cm in coarse-grained samples) exists as anhedral inclusions and subhedral/euhedral intercumulus grains. Clinopyroxene (generally less than 3.6 mm, but up to 1 cm in coarse-grained samples) exists as subhedral inclusions, anhedral poikilitic grains and/or subhedral intercumulus phases. Hornblende is a minor phase (figure 3).

The hypabyssal xenolith is a gabbro, containing phenocrysts of plagioclase and clinopyroxene with a ground-mass dominated by plagioclase and minor oxides (figure 4*f*). Plagioclase (less than 2.8 mm) is subhedral with zoning and inclusions of oxides, while clinopyroxene (less than 2 mm) is subhedral with inclusions of oxides and plagioclase. Hornblende is absent.

Canouan igneous cumulate xenoliths display numerous instances of multiple generations of the same phase (electronic supplementary material, table S3*e*). An overall scheme of crystallization sequence is proposed: olivine if present, is the first phase to crystallize, usually followed by oxides, and then plagioclase or clinopyroxene (half of the samples show that plagioclase crystallized before clinopyroxene and vice versa). This may be followed by a second generation of one or more of these phases.

#### 3.1.6. Catholic Island

One igneous cumulate xenolith was studied from Catholic Island. This sample is an anorthosite with an adcumulate texture (electronic supplementary material, table S3*f*). It is dominated by large plates of subhedral plagioclase grains with sutured contacts and interiors with sieve textures and microcracks. Olivine exists as an included minor phase.

#### 3.1.7. Carriacou

**Figure 5.**
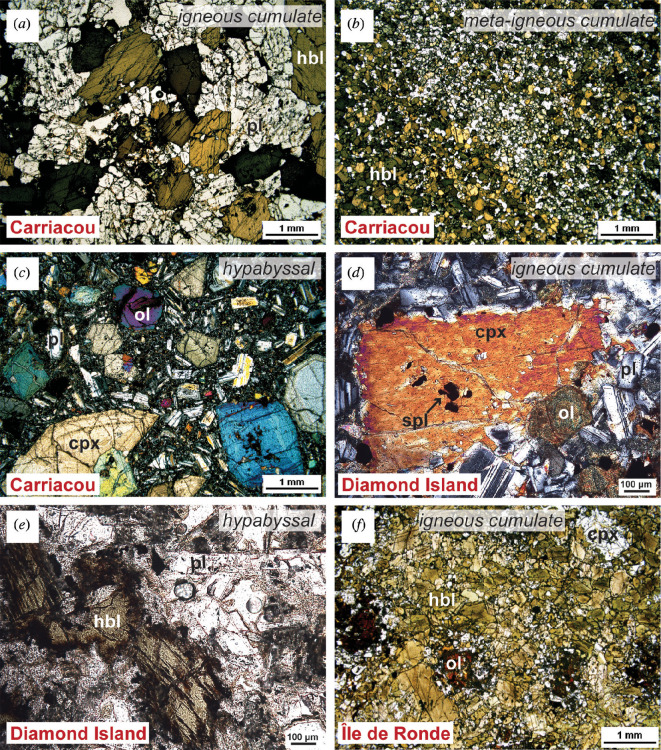
Representative photomicrographs of crustal xenolith textures. (*a*) Carriacou hornblende gabbro in plane-polarized light (ppl), igneous cumulate (CR1). Hornblende is prismatic; (*b*) Carriacou layered hornblende gabbro in ppl, meta-igneous cumulate (CR3); (*c*) Carriacou hypabyssal dolerite in cross-polarized light (xpl) (CR32). Olivine is partially iddingsitized; (*d*) Diamond Island olivine gabbro in xpl, igneous cumulate (DR4). Olivine is completely iddingsitized; (*e*) Diamond Island hypabyssal diorite in ppl (DR1); (*f*) Île de Ronde olivine and plagioclase bearing pyroxene hornblendite in ppl, igneous cumulate (RN1). Olivine (iddingsitized) is surrounded by clinopyroxene and orthopyroxene coronas.

Carriacou xenoliths exhibit all three textural subdivisions: igneous cumulate, hypabyssal and meta-igneous cumulate (electronic supplementary material, table S3*g*). Igneous cumulate xenoliths (adcumulate to orthocumulate textures) are primarily hornblende gabbros (e.g. figure 5*a*), comprising euhedral to poikilitic hornblende (less than 5 mm), euhedral to anhedral plagioclase (less than 2.5 mm), subhedral clinopyroxene (less than 1.5 mm) and oxides (less than 500 µm). Typically, clinopyroxene is rimmed by coronas of hornblende suggestive of peritectic reactions. Occasionally, anhedral iddingsitized olivine is present in the assemblage (less than 1 mm) or hornblende is absent from the assemblage, with large phenocrysts of clinopyroxene (less than 4 mm). Other igneous cumulate xenoliths are plagioclase- and/or clinopyroxene-hornblendites, where hornblende makes up greater than 90% of the sample. These samples either show subhedral hornblende, clinopyroxene and plagioclase or cumulus hornblende (less than 5 mm) (orthocumulate) with intercumulus anhedral plagioclase and clinopyroxene.

Meta-igneous cumulate xenoliths are primarily composed of inequigranular to equigranular granoblastic (olivine) hornblende- or clinopyroxene-gabbro to hornblendites. Occasionally, the transition between igneous (phaneritic) hornblende-gabbro textures with plagioclase, clinopyroxene and amphibole transitions into recrystallized equigranular granoblastic textures is visible at the thin section scale. Compositional layering is also observed (figure 5*b*). Zeolites are typically found either as thin veinlets cross-cutting the xenolith or replacing plagioclase in several meta-igneous cumulates.

Of the two hypabyssal xenolith samples, one is a dolerite consisting of mm-sized phenocrysts of partially iddingsitized olivine, clinopyroxene and plagioclase in a ground-mass of microphenocysts of plagioclase, clinopyroxene and oxides (figure 5*c*). The other is a microdiorite consisting of euhedral amphibole and plagioclase phenocrysts within a finer-grained ground-mass of clinopyroxene, plagioclase, oxides, quartz and apatite.

For igneous cumulate xenoliths, the major crystallization sequence is the early appearance of oxides, olivine and/or plagioclase (not always distinguishable), followed by clinopyroxene and hornblende. There are, however, some variations from this trend with the occasional late appearance of oxides and the second appearance of plagioclase (electronic supplementary material, table S3*g*).

#### 3.1.8. Diamond Island

Diamond Island xenoliths are igneous cumulate and hypabyssal (electronic supplementary material, table S3*h*). Igneous cumulate xenoliths are olivine gabbros (figure 5*d*) with mesocumulate textures. Oxides (less than 200 μm) are subhedral and included in all phases. Olivine (less than 2 mm) is subhedral/anhedral and partially iddingsitized. Plagioclase (less than 4 mm) is subhedral. Clinopyroxene exists either as zoned euhedral megacrysts less than 10 mm or subhedral less than 2 mm grains. All other phases show preferential alignment around clinopyroxene megacrysts suggesting they are entrained rather than grown *in situ*. A proposed crystallization sequence is the first appearance of oxides and megacrystic clinopyroxene, followed by its entrainment in a melt with a crystallization order of oxide, olivine, clinopyroxene and plagioclase.

Hypabyssal xenoliths (figure 5*e*) are phaneritic diorites with mainly similar-sized grained phases (less than 1 mm) of subhedral/anhedral oxide, plagioclase, clinopyroxene and hornblende. Phaneritic xenoliths exhibit an early appearance of oxide, followed by the co-appearance of plagioclase, clinopyroxene and hornblende.

#### 3.1.9. Île de Ronde

All Île de Ronde xenoliths are of the igneous cumulate variety (figure 5*f*), consisting of olivine and/or plagioclase-bearing hornblendites, olivine-hornblende gabbros, hornblende gabbros and hornblende and/or olivine gabbronorites (electronic supplementary material, table S3*f*). Textures are adcumulate to orthocumulate. Oxides (less than 500 μm) are subhedral/anhedral occurring only as inclusions and isolated grains in coarse-grained samples (less than 2 mm). Olivine (less than 3 mm) is completely altered to iddingsite occurring either as anhedral grains or surrounded by a clinopyroxene and/or orthopyroxene corona. Plagioclase grains (generally less than 3 mm but can be up to 8 mm in coarse-grained samples) are euhedral to anhedral and sieve textured. Some interstitial grains can be lath-like. Clinopyroxene (less than 2.8 mm) grains are subhedral to anhedral, showing uralitization. Orthopyroxene (less than 500 μm) is subhedral. Hornblende (less than 7.8 mm, but up to 2 cm in coarse samples) is either prismatic, subhedral or poikilitic with chadacrysts of oxides, iddingsite, plagioclase and/or clinopyroxene. Although variable, the predominant crystallizing sequence identified is oxides, iddingsite, plagioclase, clinopyroxene and hornblende, followed by a second generation of plagioclase. Where orthopyroxene is present in the assemblage, it appears in tandem with clinopyroxene.

### 3.2. Mineral chemistry

Full xenolith mineral analyses for Isle à Quatre, Mustique, Petite Mustique, Savan Island, Canouan, Catholic Island, Carriacou, Diamond Island and Île de Ronde (including data from Goode [86]) are presented in electronic supplementary material, table S4. In corresponding figures, published xenolith compositional data are incorporated from Bequia [15], Petit St Vincent [14] and Grenada [45] for comparison. For clinopyroxene and hornblende, Mg# is expressed as 100 Mg/(Mg + Fe^Total^). Although sulphides are present in Carriacou xenoliths, they are not described here.

#### 3.2.1. Olivine

Measurement of olivine compositions (from partially iddingsitized samples) was possible only in Canouan igneous cumulate, Carriacou hypabyssal and Diamond Island igneous cumulate xenoliths, ranging Fo_77–74_, Fo_91–70_ and Fo_86–76_, respectively (table 1). As for forsterite, NiO contents are highest in Carriacou (0.05–0.34 wt%), followed by Diamond Island (0.02–0.13 wt%) and lowest in Canouan (≤0.04 wt%), positively correlating with Fo.

#### 3.2.2. Plagioclase

**Figure 6.**
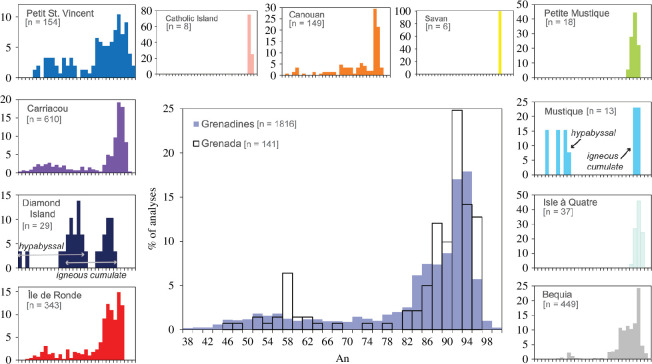
Plagioclase compositions (mol % anorthite) of crustal xenoliths in the Grenadines and Grenada. Grenadines distributions are displayed separately and combined in the middle plot for comparison with Grenada. The number of analyses for each locality is displayed in brackets. Data are taken from this study, Stamper *et al.* [45], Camejo-Harry *et al.* [15], Melekhova *et al.* [28], Aufrère [36], Camejo-Harry [46] and Melekhova *et al.* [14].

Figure 6 and table 1 summarize the variation in anorthite across studied xenolith plagioclase. Igneous cumulate xenolith plagioclases from Isle à Quatre, Petite Mustique, Savan Island and Catholic Island are all calcium rich with narrow compositional ranges (An_96–88_). Île de Ronde igneous cumulate xenolith plagioclases by contrast, while also calcic, extend to much lower An values (94–29). Mustique and Diamond Island plagioclases show a bimodal distribution in terms of An, which can be matched to igneous cumulate (An_93–90_ and An_88–65_, respectively) and hypabyssal (An_88–65_ and An_73–37_, respectively) xenolith types. Distinct An compositions are also observed for Canouan igneous cumulate and hypabyssal xenoliths (An_96–43_ and An_64–40_, respectively). Carriacou igneous cumulate, hypabyssal and meta-igneous cumulate xenolith plagioclase compositions are An_96–13_, An_90–37_ and An_96–57_, respectively. K-feldspar was measured in igneous cumulate (Or_99–63_) and meta-igneous cumulate (Or_99–92_) xenoliths from Carriacou only. Normal zoning is dominant (less than 44% rimward decreases in An) with noted incidences of reverse zoning in Petit Mustique and Île de Ronde (less than 4% rimward increases in An).

**Figure 7.**
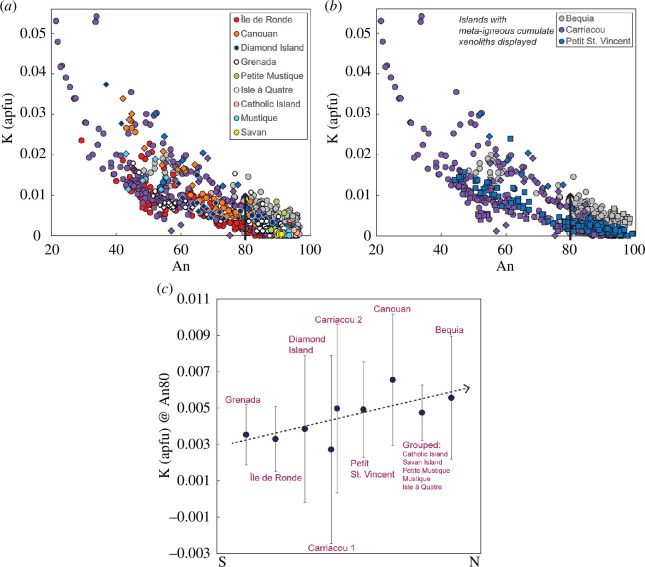
Variation in plagioclase composition in terms of potassium apfu for crustal xenoliths from the Grenadines and Grenada. Colours vary by volcanic island as shown in key. Igneous cumulate xenoliths are filled circles, meta-igneous cumulate xenoliths are filled squares and hypabyssal xenoliths are filled diamonds. (*a*) K (apfu) versus An for igneous cumulate xenoliths and hypabyssal xenoliths from all localities. (*b*) K (apfu) versus An for islands containing meta-igneous cumulate xenoliths only (Bequia, Petit St Vincent and Carriacou). Note that meta-igneous cumulate xenoliths follow similar trends to other xenolith types by location. (*c*) Results of linear regression analyses for each island distribution (including all xenolith types). Note data for Catholic Island, Savan Island, Petite Mustique, Mustique and Isle à Quatre grouped for the regression. At An_80_, calculated K (apfu) shows an overall increase from Grenada to Bequia (south to north). Carrriacou’s two diverging trends are highlighted.

For xenoliths displaying large An ranges, iron concentrations generally increase to a maximum before subsequently decreasing with increasing An. The Fe maxima vary with An for a given locality (peak Fe is 0.06 atoms per formula unit (apfu) at An_62_ for Carriacou) mirroring variations in the onset of magnetite crystallization. Potassium concentrations decrease with increasing An, with an apparent divergence into two trends (figure 7*a,b*). Carriacou xenoliths have the highest measured K of 0.22 apfu (not shown in figure 7*a,b*), followed by a more contiguous downward trend from 0.03 apfu towards higher An for all remaining localities.

#### 3.2.3. Pyroxene

**Figure 8.**
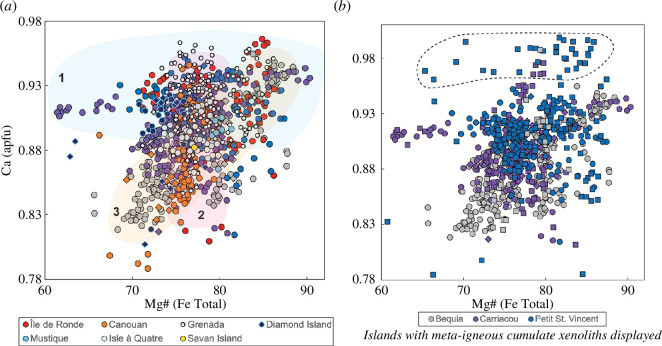
Variation in clinopyroxene compositions in terms of Ca apfu versus Mg# for crustal xenoliths from the Grenadines and Grenada. Colours vary by volcanic island as shown in key. Igneous cumulate xenoliths are filled circles, meta-igneous cumulate xenoliths are filled squares and hypabyssal xenoliths are filled diamonds. (*a*) Displays igneous cumulate and hypabyssal xenoliths only from all localities. Three broad island groupings can be distinguished: (1) Île de Ronde, Diamond Island, Carriacou and Petit St Vincent; (2) Canouan, Savan Island, Mustique and Isle à Quatre; and (3) Bequia. (*b*) Displays islands with meta-igneous cumulate xenoliths only. High Ca meta-igneous cumulate xenoliths clinopyroxenes are outlined.

Clinopyroxene compositions are predominantly diopside and Ca-rich augite. Variation in Mg# by locality is extensive and is summarized in table 1. Normal zoning is prevalent (less than 2% rimward decreases in Mg#), with fewer instances of reverse zoning observed in Isle à Quatre and Île de Ronde (less than 5% rimward increases in Mg#). Clinopyroxene macrocrysts observed in Diamond Island igneous cumulate xenoliths show oscillatory zoning. Collectively, there are no clear trends in tetrahedral aluminium, titanium or calcium (figure 8*a*) with Mg# among islands. Generally, all xenolith types overlap, with the exception of a group of high Ca meta-igneous cumulate xenoliths (figure 8*b*).

Orthopyroxene compositions range En_94–78_ (Mg#_96–79_) for Île de Ronde and En_77–76_ (Mg#_80–79_) for Savan Island.

#### 3.2.4. Hornblende

Following the classification schemes of Leake *et al.* [87], Isle à Quatre, Petite Mustique, Savan, Carriacou, Diamond and Île de Ronde hornblende compositions are magnesiohastingsite, with some Carriacou, Diamond Island and Île de Ronde xenoliths also spanning the edenite field. Mustique hornblende compositions are all edenite. The variation in Mg# by locality is summarized in table 1. Zoning is widespread (rimward decreases of less than 4% Mg# and increases of less than 5% Mg#).

**Figure 9.**
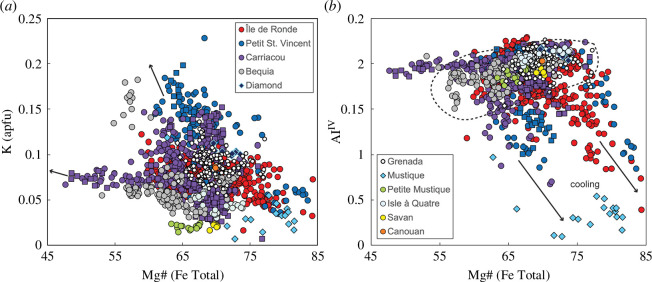
Variation in hornblende compositions in terms of (*a*) K apfu and (*b*) Al^IV^ versus Mg# for crustal xenoliths from the Grenadines and Grenada. Colours vary by volcanic island as shown in key. Igneous cumulate xenoliths are filled circles, meta-igneous cumulate xenoliths are filled squares and hypabyssal xenoliths are filled diamonds. Meta-igneous cumulate xenoliths overlap with other xenolith types in Carriacou and Petit St Vincent. In (*a*) arrows highlight the bifurcating trend for Carriacou. In (*b*) arrows denote cooling trends, while stippled line groups islands with similar distributions.

Potassium (figure 9*a*), titanium and tetrahedral aluminium (figure 9*b*) contents broadly decrease with increasing Mg#. Potassium exhibits a bifurcating trend when plotted against Mg# although hornblendes lying along both trends are found at Carriacou. Mustique hypabyssal hornblendes are distinctive in their very low Al, Ti and alkali contents.

#### 3.2.5. Spinel

**Figure 10.**
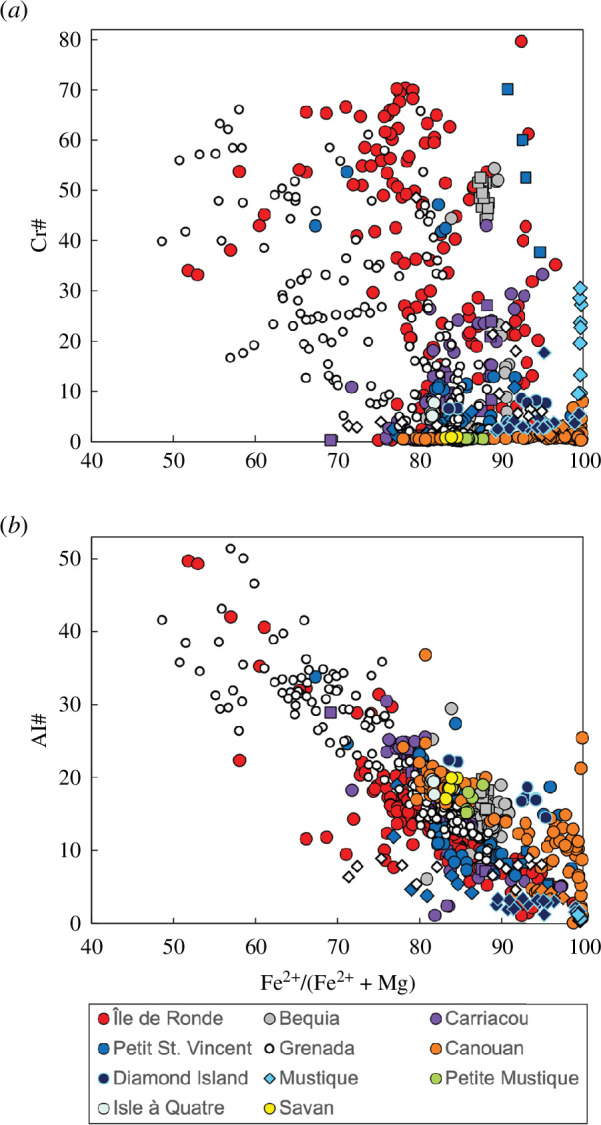
Variation in spinel composition for crustal xenoliths from the Grenadines and Grenada in terms of (*a*) Cr# and (*b*) Al# versus Fe^2+^#. Colours vary by volcanic island as shown in key. Igneous cumulate xenoliths are filled circles, meta-igneous cumulate xenoliths are filled squares and hypabyssal xenoliths are filled diamonds.

Spinel exhibits a complete continuum from chromite to titaniferous magnetite. The most Cr-rich spinel (in terms of Cr# = Cr/Cr + Al) is observed in Île de Ronde (Cr# <80) (table 1 and figure 10*a*). A few high Al_2_O_3_ (18–27 wt%) spinels, with Cr#3–43 and Fe#52–61 are present in Île de Ronde (RN1) as inclusions in an iddingsite-clinopyroxene xenocryst (figure 10*b*). Previously, pleonaste (although of intermediate composition) spinel was thought to only occur in Grenada, within the Lesser Antilles arc [16,45].

### 3.3. Southern Lesser Antilles comparisons

#### 3.3.1. Crustal xenolith textures

Primary mineral phases across Grenadines and Grenada xenoliths are similar (figure 3*a*), with differences only occurring in the restricted presence of ilmenite and orthopyroxene. The juxtaposition of incipient iddingsitized olivine (e.g. figures 4*e* and 5*c*,*d*,*f*) within an otherwise unaltered assemblage is a ubiquitous feature of the southern arc. Analyses undertaken by Brown [88] reveal the presence of iddingsite in St Vincent xenoliths, a detail previously undocumented by Tollan *et al.* [89]. However, unlike Grenada and the Grenadines, fresh, unaltered olivine is also prevalent in St Vincent xenoliths. Our recent re-examination of additional xenoliths from Grenada has uncovered a higher prevalence of zoning, mesocumulate to orthocumulate textures, uralitization, variable crystallization sequences and late-stage hornblende crystallization (interstitial and poikilitic) than reported by Stamper *et al.* [45]. This points towards pervasive metasomatic infiltration of hydrous, diverse melts and low-temperature fluids facilitating open system processes in this region of the arc [90]. Serpentinization of olivine to form iddingsite is limited to temperatures below approximately 400°C at crustal pressures [91] indicating that this feature reflects the continued reaction of originally magmatic assemblages at sub-solidus conditions in the presence of hydrous fluids.

Though there is an overall north–south increase in the proportion of mafic xenoliths (figure 3*a*), textural groupings do not appear to show any systematic trend. All islands in the Grenadines and Grenada have igneous cumulate xenoliths, while meta-igneous cumulate xenoliths have only been reported in Grenada, Carriacou, Petit St Vincent and Bequia [13–15,45]. Hypabyssal xenoliths are noted on Diamond Island, Carriacou, Petit St Vincent, Canouan, Mustique and Bequia.

#### 3.3.2. Crustal xenolith mineral compositions

When taken as a whole, phase compositions across the Grenadines and Grenada broadly overlap. The Grenadines display wide compositional ranges (in terms of An and Mg#) indicating crystallization from chemically diverse melts during their evolution [90]. Grenada xenoliths have a narrower compositional range by comparison (e.g. figures 7*a*, 8*a* and 9). Until data from more exhaustive analyses can be included from under-sampled (and indeed unsampled) islands from the Grenadines to infer otherwise, we attribute contrasting compositional ranges observed in discrete Grenadines islands, e.g. Savan Island versus Carriacou, to disparities in sampling. There are, however, exceptions to this overall commonality; we highlight these below.

Where there is more than one generation of a phase within the assemblage, compositions either do not vary considerably between the two generations or show either more mafic or evolved compositions in the later crystallizing grains. Though predominantly calcic, plagioclase compositions range An_99–37_ (figure 6), with only Carriacou recording as low as An_12_. Islands with meta-igneous cumulate xenoliths (Bequia, Petit St Vincent and Carriacou) have the highest An contents (greater than or equal to 98) (figures 6 and 7*b*). Hypabyssal plagioclases from Mustique, Canouan, Diamond Island and Grenada display lower An than igneous cumulate plagioclases (figures 6 and 7*b*). Upon closer inspection, there is an overall increase in K with An from Grenada to Bequia, most obvious in high-Ca plagioclase feldspars (An ≥ 80). We conducted regression analyses for differentiation trends displayed by each island to compare the K content at An_80_ (data for Catholic Island, Savan Island, Petite Mustique, Mustique and Isle à Quatre grouped together). Figure 7*c* illustrates this overall south-to-north increase in K, along with Carriacou’s divergent trends.

Clinopyroxene displays what appears to be three overlapping island groupings in terms of calcium content: (i) Petit St Vincent, Carriacou, Diamond Island and Île de Ronde; (ii) Canouan, Savan Island, Mustique and Isle à Quatre; and (iii) Bequia showing a positive increase in Ca with Mg# (figure 8*a*). Grenada data are scattered among all three trends. There is also a discrete group of high-Ca meta-igneous clinopyroxenes from Carriacou, Petit St Vincent and Bequia (figure 8*b*).

For hornblende, again two trends are observed for Carriacou in terms of K content (figure 9*a*), as observed for plagioclase compositions (figure 7). Although limited in samples numbers, Petite Mustique and Savan show a constant K with Mg#. While the majority of the islands (except Mustique) show a narrow variation in Al^IV^ with Mg#, trends for Petit St Vincent, Carriacou and Île de Ronde extend to lower Al^IV^ contents with increasing Mg# indicative of cooling (figure 9*b*).

Spinel compositions display an apparent north-to-south gradation in terms of primitiveness, with Grenada xenoliths dominating domains occupied by Fe# > 49, which is then overtaken by Île de Ronde (Fe# > 52), followed by Carriacou (Fe# < 69) (figure 10*a*). This north–south transition is however not continuous, as Bequia and Petit St Vincent display Cr# < 53 and < 70, respectively, despite being located north of Carriacou. The higher Al# of Île de Ronde and Grenada spinels (figure 10*b*) has been noted above.

To summarize, we note intriguing variability in phase compositions within the Grenadines and Grenada: (i) potassium contents in plagioclase show an overall increase from south to north (Grenada to Bequia) (figure 7c); (ii) three island groupings can be distinguished using the Ca content of clinopyroxene, echoing a south–north variation (figure 8a); (iii) clinopyroxenes and hornblendes from southern Grenadines islands Petit St Vincent, Carriacou and Île de Ronde display overlapping trends in terms of Ca and Al^IV^, respectively (figures 8 and 9*b*); (iv) Carriacou plagioclases and hornblendes show two distinct trends in terms of K (figures 7*a* and 9*a*); and (v) Chromites are most abundant in Grenada and Île de Ronde, with a few outliers from Petit St Vincent and Bequia (figure 10*a*).

#### 3.3.3. Lava whole-rock chemistry

Taken as a whole, subaerial Grenadines and Grenada bulk rock compositions generally overlap (figure 2). M- and C-series liquid lines of descent, geochemically distinguished by variations in calcium for Grenada [92], are also observed on other Grenadines islands (figure 2*b*). There are, however, some exceptions. Aluminium contents are higher in the Grenadines (greater than 20 wt% Al_2_O_3_) than Grenada, reflecting elevated magmatic water contents (figure 2*c*) [93]. The Grenadines has elevated sodium contents at low MgO, which is overtaken by Grenada at high MgO (figure 2*d*). Elevated potassium contents at low MgO are observed only in Canouan (2.2–6.7 wt% K_2_O, contents greater than 3 wt% not shown in figure 2*e*). Submarine Kick-’em-Jenny consistently shows a distinct liquid line of descent.

#### 3.3.4. Intensive parameters

The mineral chemistry of xenoliths was used to constrain crustal intensive parameters using the following geothermobarometers: hornblende-plagioclase (±40°C, Holland & Blundy [85]), clinopyroxene–orthopyroxene (±40°C/2.8 kbar, Putirka [94]), clinopyroxene- and hornblende-only (±57°C/2.3 kbar and ±40°C/1.6 kbar, respectively, Higgins *et al.* [95]) and magnetite–ilmenite (±36°C, Andersen & Lindsley [96]) (table 2). Major element data for touching rims and/or included phases and their hosts were used in Île de Ronde hornblende-plagioclase and Canouan magnetite–ilmenite calculations. Outside of this, all combinations of grain pairs in each sample were used for remaining calculations and the results were averaged. Hornblende-plagioclase thermometry is limited to plagioclase less calcic than An_90_, therefore only temperatures calculated using An < 90 are reported (a requirement not met by Isle à Quatre, Savan Island and Canouan xenoliths). Because pressure has been shown to have a modest dependence on thermometry (±72°C GPa-1) at sub-volcanic pressures [98], a nominal value of 500 MPa was used where a pressure estimate was needed for temperature calculations. Temperature estimates for coexisting oxides were made using the ILMAT program of Lepage [99] with the Lindsley & Spencer [97] method for recalculation.

**Table 2.** Thermo- and oxy-barometry for xenoliths from the Grenadines and Grenada.

locality	suite	sample	method	phases	T (°C)	P (kbar)	log fO_2 relative to ΔNNO_
Bequia	igneous cumulate	BQ1	Higgins *et al*. [95]	cpx	983–999	3.7–4.7	—
		BQ4			976–1000	2–4	—
		BQ7			999–1019	2–2.9	—
		BQ8			999	4.3	—
		BQ11			985–1000	3–4.3	—
		BQA7			981–1090	2–5.4	—
		BQA13			975–976	2.6–3	—
		BQA18			964–1008	3.8–5.8	—
		BQA40			985–1026	2–4.1	—
		BQB9			999–1020	2–3.3	—
		BQB11			983–1083	1.2–4.8	—
		BQA24			914–1003	2.1–8	—
	meta-igneous cumulate	BQA3			982–1015	2–5.1	—
	hypabyssal	BQA35			1003–1025	2.1–4	—
	igneous cumulate	BQ7		hbl	940–950	2–4	—
		BQ8			948–975	2.3–5.2	—
		BQ11			962–974	2.5–4.2	—
		BQA7			937–957	2	—
		BQA10			850–942	2.5-–.3	—
		BQA18			917–995	2–5.8	—
		BQA22			883–950	4–5	—
		BQA40			920–965	2–3.2	—
		BQB11			970–998	2–2.9	—
		BQA24			781–944	2.1–4.5	—
Isle à Quatre	igneous cumulate	IQ1	Higgins *et al*. [95]	cpx	983–1007	2.8–4.3	—
		IQ2			982–1000	2–3.9	—
		IQ3			1004–1033	2.7–4	—
		IQ1		hbl	982–1019	2–3.9	—
		IQ2			959–979	2.8–4	—
		IQ3			985–1003	4.5–4.7	—
Mustique	igneous cumulate	MST1	Higgins *et al*. [95]	cpx	999–1020	2–3.7	—
	hypabyssal	MST2		hbl	777–795	2.2–2.9	—
		MST4			767–825	2.2	—
		MST4	Holland & Blundy [85]^b^	hbl-plag	798–809	assumed 5	—
Petite Mustique	igneous cumulate	PMST1.1	Higgins *et al*. [95]	hbl	917–972	2.4–4	—
		PMST1.2			956–972	2.8–4	—
		PMST1.1	Holland & Blundy [85]^b^	hbl-plag	1023–1029	assumed 5	—
		PMST1.2			1025		—
Savan	igneous cumulate	SA1	Higgins *et al*. [95]	cpx	998	3.4	—
				hbl	958–966	2.1–3.5	—
			Putirka [94]	cpx-opx	924	1.8	—
Canouan	igneous cumulate	CN5	Andersen & Lindsley [96]^a^	spl-ilm	813–825	–	1.09–1.16
		CN8			813–884	–	0.06–0.48
	igneous cumulate	CN1	Higgins *et al*. [95]	cpx	956–994	2–3.8	—
		CN2			982–1000	2–4.7	—
		CN3			1000–1024	3.5–4.6	—
		CN7			1000–1073	4–7	—
		CN10			1000–1016	3.8–4.7	—
	hypabyssal	CN5			912–1020	2–3.7	—
	igneous cumulate	CN10		hbl	969–973	2	—
Petit St Vincent	igneous cumulate	PSV1	Higgins *et al*. [95]	cpx	997–1024	2.2–4.7	—
		PSV3			1010–1100	2–4.3	—
		PSV5			985–1042	4.1–5.4	—
		PSV12			944–1000	3.1–5.5	—
		PSV14			982–1057	2.8–4.2	—
		PSV19			983–1035	3–5	—
		PSV20			988–1025	3.2–4.7	—
	meta-igneous cumulate	PSV6			975–995	3.1–5	—
		PSV8			950–1028	2.7–4.5	—
		PSV10			971–1218	2.3–8.8	—
		PSV16			1024–1224	2.3–10.4	—
		PSV17			974–1024	2–4	—
		PSV18			1021–1079	1.8–4.5	—
	hypabyssal	PSV13			1000–1025	2.2–5	—
	igneous cumulate	PSV1		hbl	915–988	2–5	—
		PSV3			958–1031	4.3–6.8	—
		PSV5			979–1000	5–7	—
		PSV12			900–968	2–7	—
		PSV14			944–974	4–5	—
		PSV19			938–966	2.3–5.7	—
		PSV20			946–991	4–6.3	—
	meta-igneous cumulate	PSV6			794–867	2.1–2.7	—
		PSV8			856–920	2–4.3	—
		PSV10			806–919	2.2–10	—
		PSV17			808–890	2.5–3.8	—
		PSV18			800–1000	2–8	—
Carriacou	igneous cumulate	CR1	Higgins *et al*. [95]	cpx	925–970	5.3–7.1	—
		CR2			903–958	5.9–7.4	—
		CR6			989–1054	3.4–8.1	—
		CR9			1029–1074	3.4–8.5	—
		CR11			1002–1088	2.3–5.3	—
		CR12			934–950	5.1–5.5	—
		CR14			989–1100	2–7	—
		CR16			984–1122	2.3–4.5	—
	hypabyssal	CR3			938	7.5	—
		CR32			1000–1082	2–9.5	—
		CR36			1005–1033	2–4.3	—
	meta-igneous cumulate	CR3			925–977	5–7.5	—
		CR25			1011–1071	2–7	—
		CR26			982–1020	2–5	—
		CR34			997–1012	2–4.1	—
	igneous cumulate	CR1		hbl	887–948	4.1–6.2	—
		CR2			892–950	5–8	—
		CR5			776–975	2.2–4.7	—
		CR6			900–980	4.4–5	—
		CR7			790–906	2.4–4.5	—
		CR9			939–1024	4.2–9.2	—
		CR10			880–948	2.1–5	—
		CR11			879–955	2.5–4	—
		CR12			900–990	4–7.2	—
		CR16			942–981	3.5–5	—
		CR22			778–967	2.1–4	—
		CR26			950–960	5	—
		CR29			951–1000	2–4.7	—
		CR38			975–990	4.2–5	—
		CR39			969–1008	2–6	—
		CR49			950–962	2.4–4	—
		CR58			984–1000	4–5.3	—
		CR62			958–985	2–4	—
		CR63			974–998	2–4.7	—
	meta-igneous cumulate	CR3			887–950	4.5–7	—
		CR8			888–950	3.9–8	—
		CR25			925–958	5	—
		CR26			940–961	5	—
		CR34			966–980	2–4.2	—
		CR35			940–962	2–2.8	—
		CR37			965–990	3.5–5	—
		CR42			968–1000	3–4.5	—
		CR46			958–965	3–4.7	—
		CR56			965–986	2–4.5	—
		CR59			959–970	2.5–4	—
		CR66			915–1038	2.1–6	—
	igneous cumulate	CR11	Holland & Blundy [85]^b^	hbl-plag	809–985	assumed 5	—
		CR12			861–934		—
		CR16			926–978		—
		CR22			766–897		—
		CR63			901–1016		—
	meta-igneous cumulate	CR37			985–1015		—
		CR46			962–996		—
		CR56			923–961		—
		CR66			904–994		—
Diamond Island	igneous cumulate	DR2	Higgins *et al*. [95]	cpx	1011–1040	3.5–6.2	—
		DR4			1000-1033	4.2–8.3	—
	hypabyssal	DR1			1001–1046	3.7–6.2	—
		DR5			925–976	2.3–6	—
		DR1		hbl	875–1000	3–5	—
		DR1	Holland & Blundy [85]^b^	hbl-plag	823–895	assumed 5	—
		DR5			895		—
Île de Ronde	igneous cumulate	RN5	Holland & Blundy [85]^b^	hbl-plag	838	assumed 5	—
		RN7			928–985		—
		RN13			897–952		—
		RN16			888–931		—
		RN6			957–966		—
		RN8			924–957		—
		RN1	Higgins *et al*. [95]	cpx	925–1091	2.1–3.3	—
		RN6			950–974	3–4.3	—
		RN7			974–975	2.5–2.7	—
		RN8			956–984	2.8–4.8	—
		RN11			970–1100	2.5–6.4	—
		RN12			940–1044	2.8–5.9	—
		RN13			908–1067	3–4.9	—
		RN16			926–1026	4–5	—
		RN18			1005–1094	2–4.1	—
		RN1		hbl	898–1028	3.4–8	—
		RN5			848–930	2.2–5.3	—
		RN6			926–985	2.5–6.3	—
		RN7			880–960	2.6–8	—
		RN8			923–960	2.1–3.3	—
		RN11			926–1011	2–5.3	—
		RN12			950–1003	3.2–7.2	—
		RN13			867–945	2.5–5	—
		RN16			860–948	2.5–4.5	—
		RN18			845–1020	3–9.2	—
		RN11	Putirka [94]	cpx-opx	898–902	3.5–3.9	—
		RN23			804	1.9	—
		RN20			834	2.4	—
		RN21			793–818	4–4.2	—
Grenada	igneous cumulate	GR2	Higgins *et al*. [95]	cpx	969–1100	2–4.6	—
		GR4			1000–1093	1.8–6	—
		GR5			965–1092	2.3–4.6	—
		GR6			976–1009	3–4.7	—
		GR9			932–976	4–6	—
		GR15			996–1079	1.8–4.5	—
		GR17			1000-1075	2–5.6	—
		GR21			982–1079	3.4–5	—
		GR24			1001–1087	4.2–5	—
		GR25			1025–1040	3.4–4.2	—
		GR29			1033–1110	3.8–5	—
		GR33			975–1012	2–5	—
		GR36			1068–1100	2.1–5	—
		GR40			995–1115	2–5.6	—
		GR42			949–982	2.3–3	—
		GR59			986–1040	4-5	—
		GR2		hbl	946–978	2–3.6	—
		GR4			965–980	2.7–4.5	—
		GR5			952–1012	2.5–5	—
		GR6			981–962	4.7–5	—
		GR11			960–1000	2–6.9	—
		GR15			940–964	2–4	—
		GR17			964–1016	2.6–7.1	—
		GR21			950–1010	2–5.8	—
		GR24			959–1000	2–5.3	—
		GR25			984–1023	4.8–5.5	—
		GR29			1000–1024	4.3–5.3	—
		GR33			950–962	2–5	—
		GR40			952–980	2–4.5	—
		GR42			875–961	2–4.6	—
		GR52			962–980	2.7–5	—
		GR59			972–980	3.5–5	—

*Notes:* Intensive parameters for Grenada, Petit St Vincent and Bequia xenoliths were calculated using published major element data [14,15,45].

^a^ Lindsley and Spencer [97] oxide formula recalculation.

^b^ Edenite + albite = richterite + anorthite.

Xenolith crystallization pressures (1.2–10.4 kbar) and temperatures (623–1224°C) are extensive across the Grenadines and Grenada reflecting varying spatial and temporal crustal conditions. Crystallization pressures appear to systematically increase from Grenada to Petit St Vincent (electronic supplementary material, figure S1*a*). Pressure ranges in Canouan and Bequia are also extensive, which contrasts with Savan Island, Petite Mustique, Mustique and Isle à Quatre. This could be interpreted as a reflection of sampling biases; however, it is worth noting the similarity in sample numbers studied for Diamond Island (electronic supplementary material, table S3) which displays a broader pressure range (electronic supplementary material, figure S1*a*). Hypabyssal xenoliths generally display shallower pressures (2–5 kbar) than their igneous and meta-igneous cumulate counterparts. Excluded from this are Bequia igneous cumulate xenoliths displaying the lowest pressures (1.2–1.7 kbar) for this arc segment and two high-pressure analyses obtained for Carriacou hypabyssal xenoliths (7.5 and 9.5 kbar). Intriguingly, Diamond Island hypabyssal xenoliths show a broad pressure range mirroring igneous and meta-igneous cumulate xenolith pressure ranges for other nearby islands. Where present, meta-igneous cumulate xenolith pressures tend to overlap with igneous cumulate xenoliths. Petit St Vincent meta-igneous cumulate xenoliths have the highest pressures for the entire suite.

There are no clear trends north to south in terms of average crystallization temperatures by thermometer (electronic supplementary material, figure S1*b*). Islands with multiple xenolith types exhibit broadly overlapping temperature ranges. One exception is Mustique with clear low and high temperatures for hypabyssal and igneous cumulate xenoliths, respectively. Petit St Vincent is another exception with meta-igneous cumulate xenoliths displaying discrete low (790–905°C) and high (1207–1224°C) temperatures separate from their overlapping trend with igneous cumulate and hypabyssal xenoliths. This distinction is related to the high proportion of Petit St Vincent meta-igneous cumulate xenoliths containing striking deformation microstructures when compared with the rest of the arc advocating for recrystallization involving fluid/melt interaction [14].

**Figure 11.**
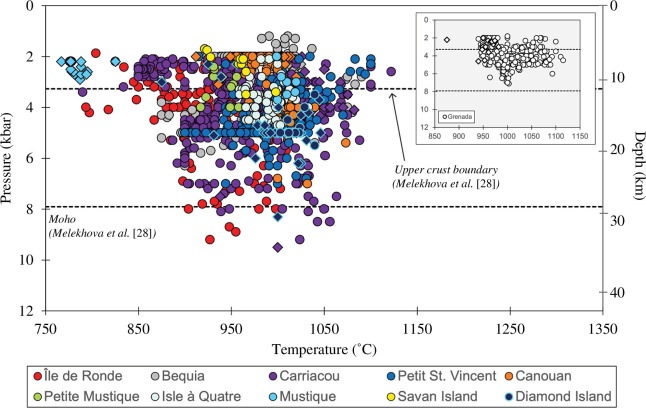
Temperature versus pressure variation of crustal xenoliths from the Grenadines using estimates from clinopyroxene- and hornblende-only thermobarometer of Higgins *et al.* [95] and clinopyroxene–orthopyroxene thermobarometer of Putirka [94]. Data are divided by xenolith type: igneous cumulate xenoliths are filled circles and hypabyssal xenoliths are filled diamonds (meta-igneous cumulate xenoliths are excluded). Grenada and St Vincent (calculated using data from Tollan *et al.* [89]) data are shown for comparison as an inset (note the different temperature scale). Stippled lines denote depths to the upper crust and Moho beneath Grenada and St Vincent after Melekhova *et al.* [28]. Note the conspicuously narrower crystallization pressure and temperature ranges for Grenada and St Vincent compared with the Grenadines. This figure is adapted from Higgins and Caricchi [4].

To gain further insight into crustal process, we elucidate further trends in pressure–temperature space (figure 11). The first observation is that the Grenada suite of xenoliths, despite having a higher occurrence of ultramafic compositions (wehrlites, pyroxenites and hornblendites), are extracted from shallower pressures than Grenadines xenoliths when taken as a whole. This contrasts with greater depths collated for Grenada xenoliths by Melekhova *et al.* [28] estimated by phase equilibrium experiments. This pattern of mafic upper/mid crust, however, is also emulated in the Grenadines, with the occurrence of hornblendites, mela gabbros, mela gabbronorites and olivine gabbros. More evolved rock types are also present in the upper/mid crust (leuco gabbros, gabbros and gabbronorites in the Grenadines; gabbros in Grenada) lending to the idea of lateral variability in the crust. In the Grenadines, compositions of xenoliths dominating the lower crust are plagioclase hornblendites and gabbros (olivine-free), with minor instances of olivine gabbros, leuco gabbros and gabbronorites. This trans-crustal variability can be supported by the polybaric differentiation of chemically diverse melts and gives rise to the variable crystallization sequences and multiple phase generations observed in the Grenadines.

Oxygen fugacity calculations were only possible for Canouan xenoliths, ranging 0.06–1.16 relative to the NNO buffer. Such oxidizing conditions have also been reported for Grenada, Petit St Vincent and Bequia xenoliths [14,15,45].

## 4. Discussion

### 4.1. The magma–mush paradigm in the southern Lesser Antilles

Before considering the petrological trends elucidated by our findings, it is important that we inspect existing concepts surrounding the architecture and dynamics of plumbing systems within the southern Lesser Antilles. The Grenadines are bracketed by larger islands Grenada (south) and St Vincent (north), each distinguished by a deeper magma source in mantle wedge beneath Grenada (garnet-bearing peridotite) than St Vincent (spinel-bearing peridotite) [45]. Eruption temperatures of primitive basalts from St Vincent (1130–1180°C; Heath *et al.* [100]) and Grenada (1325°C; Stamper *et al.* [45]) match this increase in melt generation depth. The overall north–south increase in the mafic character of xenoliths in the Grenadines and Grenada can be linked to these differences in parental magma compositions for St Vincent and Grenada [45]. Anomalously low eruption temperatures from Kick-’em-Jenny primitive basalts (approx. 1000°C [37]), however, emphasize the need to be cautious when generalizing trends along the arc largely because of the effect of H_2_O on lowering the liquidus temperature. It remains to be seen how Kick-’em-Jenny’s atypical primitive magmas manifest in terms of xenolith character because no xenoliths have yet to be recovered from this volcano.

Studies of crustal xenoliths in the Lesser Antilles have promoted the existence of vertically extensive crystal-rich mushes dominating sub-volcanic systems [14,17,28,46,101]. We have shown that xenoliths in the Grenadines and Grenada display a range of textures and compositions indicative of crystallization over a wide range of pressure–temperature–melt composition conditions (figure 11). In the upper crust (less than 3.3 kbar, depth to layer 2 of crustal structure beneath Grenada to St Vincent proposed by Melekhova *et al.* [28]), xenoliths collectively record a broader temperature range compared with greater depths. This aligns with the crustal thermochemical stratification previously identified by Higgins & Caricchi [4]. Within crustal hot zones, polybaric differentiation involves percolative reactive flow of melt through the mush. Buffering by low-variance mineral assemblages in mid/lower crustal regions generates melts of a comparatively narrow compositional range [90], while further differentiated evolved melts ascend and stall in the upper crust [4,42]. The upward migration of interstitial melts is facilitated by buoyancy and deformation-assisted compaction [102]. The existence of meta-igneous xenoliths across this region, combined with their location throughout the crust (electronic supplementary material, figure S1) suggests that deformation plays an important role in melt expulsion.

It is, however, difficult to resolve inter-island differences in magma storage despite our attempts to populate the gap in xenolith data for the Grenadines. This is because there remains an absence of data from the majority of the Grenadines (more than 32 islands make up the archipelago), combined with sampling disparities between studied islands and biases of igneous cumulate over hypabyssal and meta-igneous cumulate xenoliths [4]. Despite these limitations, our thermobarometry estimates reveal interesting variations in crustal processes from Grenada to Bequia. Grenadines islands Île de Ronde, Diamond Island, Carriacou, Petit St Vincent and Bequia all display higher xenoliths crystallization pressures than Grenada (Figure 11 and electronic supplementary material, figure S1) pointing towards more extensive trans-crustal magmatic systems beneath these Grenadines islands, facilitating protracted cooling and crystallization.

It is curious that out of all the Grenadines, Kick-’em-Jenny is the only volcanic centre exhibiting present-day eruptive activity. Moreover, adjacent Grenada has attained a size suggestive of long-lived magma storage systems [15], albeit with no historical eruptions. Results of our textural analysis of xenoliths (widespread zoning, mesocumulate to orthocumulate textures, uralitization, variable crystallization sequences and late-stage phase crystallization) demonstrate that porous reactive flow prevails in crystal mushes beneath the Grenadines and Grenada. The crystal-rich nature of mush zones makes them inefficient at cooling validating long thermal lifetimes [68]. However, this can only be sustained by replenishment of mantle-derived basalt from below, which in turn modulates the volume of erupted material [103–105]. Systems that lack basaltic input cannot survive for long and may cease to support surface volcanism [68]. In the following sections, we explore tectonic and structural conditions for variations in the formation of eruptible, cogenetic magma bodies.

### 4.2. Geophysical and tectonic considerations

Magma genesis and the positioning of volcanic arcs within subduction zones are governed by (i) the delivery of water to the mantle wedge from hydrated minerals bound to the downgoing slab (crust, sediment and mantle lithosphere) lowering the solidus sufficiently to promote melting of the overlying mantle; (ii) thermal conditions within the mantle wedge controlling where melting occurs; and (iii) pathways for melt/fluid migration [106–110]. In the Lesser Antilles, serpentine residing in subducted fracture zones has been proposed as the dominant supplier of water to the wedge [9]. There are fracture zones located near the boundary between older Proto Caribbean and current Equatorial Atlantic domains (central arc segment) and Grenadines/Grenada (figure 1). The highest concentrations of fluids and melt are found below Guadeloupe, Dominica and Martinique, as inferred by low-velocity anomalies in the mantle wedge [25,26], resulting in these islands having had the highest volume of erupted magma over the last 100 000 years [111]. The inferred presence of excess fluids/melt beneath Grenada/Grenadines probably supplied by the Proto-Caribbean fracture zone (figure 1) [25,26] has not manifested in similar levels of observed magmatic productivity. Plausible contributors lie in the quantity and source of fluids added to the mantle in the southern arc (excluding St Vincent) compared with the central arc. Using boron isotope data, Cooper *et al.* [9] show that fluid inputs beneath Grenada and the Grenadines are low and derived from altered oceanic crust and sediment, not serpentine.

There are a complexity of factors influencing wedge dynamics [107,112] far beyond the scope of this article. One candidate is the difference in depth to the slab surface along arc strike. In the Lesser Antilles, this is shallowest beneath Grenada and the Grenadines [113,114]. Syracuse & Abers [115] put forward the suggestion that larger slab depths may correspond to a thicker mantle wedge facilitating higher degrees of flux melting. Following with this reasoning, a ‘choked’ mantle wedge in the southern arc could narrow its temperature structure restricting melt migration to the upper plate.

A third consideration relates to recent findings for fluid and melt pathways beneath the Lesser Antilles by Hicks *et al*. [114]. These authors find that rather than rising vertically from the subducted slab through the mantle wedge and upper plate to the arc above, melts and fluids are carried further downwards in the mantle on a trajectory towards the back-arc. Melt then pools at the base of the overriding plate below the back-arc before being transported back towards the volcanic arc through the crust. Overriding plate structure therefore exerts an important control on melt percolation to the surface. Hicks *et al.* [114] suggest preferential channelling through pre-existing extensional structures formed during back-arc spreading, upon which the present-day Lesser Antilles arc is built [22]. However, it is unclear how the thickened crust and absence of back-arc spreading north of Dominica noted by Allen *et al.* [22] fit into this hypothesis. The stress regime of the upper plate has been shown to control magma transport and the distribution of volcanoes [116]. For the Grenadines and Grenada, perhaps additional stresses introduced by the transpressional interaction between the Southern Caribbean plate boundary zone and South America [52] (figure 1) encumbers melt transfer through the upper plate.

The bathymetric delineation of the SLAAP (figure 3*b*) points towards a shared history of uplift for the Grenadines and Grenada. Speed *et al.* [19] propose that this uplift required inflation supplied by an intrusive phase of arc magmatism. In the following paragraphs, we examine the applicability of two mechanisms for this regional uplift.

At face value, several observations support the process of delamination as a possible mechanism for uplift in the Grenadines and Grenada, whereby dense arc crust sinks or founders into the underlying mantle, allowing the upwelling of hot material at the base of the crust [117,118]. These are (i) presence of mafic crustal xenoliths (figure 3*a*) together with noted mantle xenoliths from Grenada [119]; (ii) evidence for the reworking of arc crust from igneous and meta-igneous xenoliths in the presence of fluid and H_2_O-rich melt [14]; (iii) prevalence of primitive magmas absent from central and northern arc segments (figure 2); and (iv) a focused, low-velocity anomaly in the mantle wedge beneath the Grenadines interpreted as recording fluid flux, upwelling and/or partial melt [25,26]. Experiments have shown that hydrous magmas have a tendency to form dense, garnet-bearing ultramafic plutonic rocks at lower crustal conditions, which are always denser than upper mantle rocks [120]. However, garnet is not a constituent of crustal xenoliths erupted in the Lesser Antilles, and mantle xenoliths from Grenada have been shown to have similar densities to their ultramafic crustal equivalents, approximately 3.3 g cm^−3^ [28]. An added complication is that despite the overall gradual increase in seismic P wave velocity (*V*_P_) with depth (positively correlated with xenolith densities) within the Lesser Antilles arc crust, there is a distinct low-velocity mid-crustal layer under Grenada and the Grenadines [28]. In the absence of gravity data for the southern Lesser Antilles to provide additional constraints, a case for delamination is insubstantial.

A second potential mechanism causing the uplift of Grenada and the Grenadines is the localized development of hot regions in the mantle wedge. A recent study by Braszus *et al.* [10] identified a lateral tear 200 km depth in the subducting slab beneath Grenada interpreted to have developed along an inferred Proto-Caribbean fracture zone between 50 and 35 Ma. Such ruptures have been known to create pathways for mantle upwelling [121], providing a credible source for the repeated magma injection into the crust and subsequent regional uplift.

### 4.3. A shared plumbing system beneath the Grenadines and Grenada?

A common trait of Lesser Antilles volcanic islands is that they are constructed from eruptions of several juxtaposed volcanic centres (excluding the island of Saba which is regarded as a single stratovolcano) [41]. While this may suggest comparable conditions geographically for magma extrusion, it does not necessarily translate to a shared magmatic connection between neighbouring volcanic systems. Volcanic centres often exhibit northward or southward migrations in volcanism with or without temporal overlaps in activity (e.g. Montserrat [122]). For the island of Dominica, it has been suggested that its nine active volcanoes are fed by an island-wide batholith [123], however, evidence for such connectivity is still the subject of debate. Given its inter-island proximities and tectonic and eruptive similarities, it is conceivable for the Grenadines archipelago to embody a shared volcanic complex. Geochemical trends based on major element compositions are commonly used to band samples from similar volcanic systems. However, the compositional variability identified in crustal xenolith phases between Grenadines islands in this study suggests otherwise.

Linkages to the Grenadian volcanic system have been made based on the presence of M- and C-series magmas in the Grenadines. White *et al.* [13] assign Isle de Caille (island immediately north of Grenada) lavas to the M-series, while Melekhova *et al.* [14] implicate M- and C-series magmas in crustal growth beneath Petit St Vincent. Figure 2 illustrates that other Grenadines islands follow M- and C-series trends. However, we note that clear associations to Grenadian magma series are not ubiquitous in the Grenadines, as White *et al.* [13] found that volcanic rocks from Union Island are only intermediate between the M- and C-series.

Variations in the size and distribution of volcanic centres between the subaerial Grenadines and Grenada point towards a divergence in magmatic processes operating at the terminus of the arc. The largest Grenadines island (Carriacou) has a size of approximately 37.7 km² and the smallest (Petit St Vincent) approximately 0.52 km^2^; Grenada exceeds this with a size of approximately 344 km². Examining the spacing between the larger active volcanic islands of the Lesser Antilles (Guadeloupe to St Vincent) reveals an average distance of approximately 40 km, while the distance between Grenada and St Vincent is approximately 110 km. In contrast, the spacing between individual Grenadines islands and Grenada can be as little as less than 1 km. We suggest that these variations result from differences in magma supply rates and maturities of the plumbing systems beneath Grenada and subaerial Grenadines. This is reminiscent of submarine volcanoes from the Mariana intra-oceanic arc where it has been suggested that their size and spacing are related to their maturity: large, widely spaced volcanoes are more mature than small, closely spaced centres [124]. Bloomer *et al.* [124] go on to speculate that volcanism initiates as small irregularly spaced centres that grow together into ridges later reorganizing into a system of fewer more evenly spaced conduits. For the southern Lesser Antilles, we surmise that the identified slab tear beneath Grenada [10] drives a similar type of reorganization. Grenada’s proximity to this source of mantle upwelling promotes the channelization of magma through centralized conduits [19]. The consequent establishment of a long-lived magmatic system beneath Grenada is coincident with what we term a ‘hoovering of melt’ from nearby underdeveloped Grenadines plumbing systems due to their close proximity.

Though related geographically to Grenada and the Grenadines, Kick-’em-Jenny submarine volcano presents a distinction in having conspicuously dissimilar eruption histories and chemistries for which structural features can be invoked. Grenada and the subaerial Grenadines all reside on the SLAAP [19], while Kick-’em-Jenny is located on the eastern slope of the Grenada Basin [125] (figure 3*b*), a fact previously noted by White *et al.* [13]. We agree with White *et al.* [13] that Kick-’em-Jenny is a distinct volcano unrelated to Grenada. In terms of eruptive behaviour, Kick-’em-Jenny has a closer connection to La Soufrière, St Vincent. However, its atypical low-temperature mafic magmas imply unresolved differences in tectonics and/or thermal/compositional structure of the mantle wedge and subducted slab.

### 4.4. Arc crust preservation in the Grenadines

As depicted in earlier sections, the establishment of the modern-day active Lesser Antilles arc in the Miocene was followed by the cessation of volcanism in the southern Grenadines by the end of the Neogene. Although volcanic activity is thought to have been mostly intrusive [19], it is plausible that with the uplift of the SLAAP, any original topography would have eroded over time. We note here the observation by Melekhova *et al.* [14] that Petit St Vincent, Petite Martinique and islets Petite Dominique, Fota and Umbrella form a ring-like archipelago that could have once been part of a much larger submerged volcanic edifice. There is also remarkable diversity in erupted magma compositions among Grenadines islands spanning mafic to felsic, almost encompassing that of the rest of the arc (figure 2). Using these attributes, we speculate below that the Grenadines archipelago represents the early onset of subduction following the final migration of the arc to its present position.

Prior to this (approx. 40 Ma), magmatism was located to the east on islands making up the Limestone Caribbees (figure 1), with a new model proposing its southern extension parallel to the current arc but buried beneath the Barbados accretionary prism [22]. Around 25 Ma, the arc migrated west to its current location [10]. Volcanism would have initiated as a series of small underwater eruptions forming submarine volcanic centres. Although modern submarine volcanoes can now be observed from the imagery of the seafloor, ancient submarine rock successions are only exposed during tectonic uplift [126]. We find evidence for seawater interaction in the form of zeolites and pillow lavas on constituent Grenadines islands (e.g. electronic supplementary material, figure S2). The presence of intensely deformed meta-igneous xenoliths on Petit St Vincent [14], the likes of which have thus far not been identified elsewhere along the arc, is further validation for the exposure of reworked older arc crust provided by the uplifted Grenadines. We suggest that the lack of more occurrences of intensely deformed xenoliths in the Grenadines is due to sampling biases.

Comparisons can be drawn with other intra-oceanic subduction systems, for example, the central segment of the Kermadec–Tofua arc. Here, there are numerous, submarine volcanic centres exhibiting a broad range of lava compositions (basalt to rhyolite) [127]. In this case, young active centres are bordered to the north and south by older (active) volcanic islands. However, we can draw parallels to the physical and chemical features characterizing the initiation of contiguous volcanic activity within an arc setting. We suggest that for the Lesser Antilles arc, the uplift of the SLAAP provides rare modern insight into similar past phenomena in the Grenadines. Future detailed studies dating volcanic rocks from the Grenadines will help confirm this theory.

## 5. Conclusion

We have explored parallels in magmatic processes within the southern Lesser Antilles using insight from the petrology of erupted material and existing geophysical constructs, answering our opening research questions below:

1. The subaerial Grenadines presents the following characteristics that may be construed as resulting from a shared magmatic feeding system: absent modern-day volcanism ceasing in the Neogene with remnant small constituent volcanic islands having no edifices and flat topographies. However, our analysis of crustal xenoliths from islands covering the length of the archipelago reveals textures indicative of crystallization over a wide range of pressure–temperature–melt composition conditions in the crust (figure 11). This is facilitated by percolative reactive flow through vertically extensive crystal mushes resulting in polybaric differentiation and the formation of plutonic assemblages with variable crystallization sequences ranging from ultramafic to anorthosite. Xenolith mineral phases generally display discrete compositional trends between islands, most notably the bifurcating pattern of Carriacou (figures 7*a* and 9*a*). There are few commonalities: (i) potassium contents of plagioclase broadly increase from south to north (figure 7*c*) and (ii) clinopyroxene and hornblende compositions from southern islands Île de Ronde, Carriacou and Petit St Vincent tend to overlap (figures 8*a* and 9*b*). Overall, this points towards significant inter-island variability in crustal processes probably stemming from discrete plumbing systems.
2. Despite their bathymetric delineation, a shared plumbing system between Grenada and the subaerial Grenadines is not clear cut. On the one hand, the prevalence of M- and C-series magmas is suggestive of a common magmatic origin. On the other hand, we identify the following along-arc variations: (i) increasing mafic character of crustal xenoliths from north to south (figure 3*a*); (ii) disproportionate levels of surface activity over time manifesting in divergent island sizes between the Grenadines and Grenada; and (iii) narrower xenolith crystallization pressures and temperatures beneath Grenada than the Grenadines (figure 11). We suggest the former is related to the increasing depth of melt generation and associated eruption temperatures identified between St Vincent and Grenada [45,100].

We speculate that the Grenadines represent the early onset of subduction forming the modern-day Lesser Antilles arc. The compositional variety of erupted magmas is reminiscent of the entire Lesser Antilles (figure 2). Volcanism would have initiated as a series of small underwater eruptions forming submarine volcanic centres, confirmed by the existence of zeolites and pillow lavas on constituent Grenadines islands (e.g. electronic supplementary material, figure S2). Subsequent uplift of the SLAAP would have eroded the original topography of these volcanoes over time with the eventual cessation of volcanism. The positioning of the Grenadines on an elevated platform provides rare modern insight into early arc crust formation not commonly preserved in established arcs.

We propose that Grenada’s development on the SLAAP was fundamentally different from the Grenadines due to the presence of a lateral slab tear beneath Grenada [10] creating a pathway for mantle upwelling. This would have triggered patterns of surface volcanism not observed in the Grenadines, allowing Grenada to attain a size suggestive of a long-lived magma reservoir. The observed north-to-south progression in the mafic character of xenolith mineral assemblages (figure 3*a*) and spinels (figure 10*a*) is probably a manifestation of the proximity of volcanic centres to this source of mantle upwelling. The Grenadines' shared inclination for comparatively short-lived volcanism can be explained by what we describe as a ‘hoovering of melt’ towards the well-established plumbing system of nearby Grenada. Other tectonic features may have also hindered the sustained accumulation of eruptible magma reservoirs in the crust such as (i) shallow subducted slab depths limiting the thermal flux required for slab dehydration and melt generation and (ii) overriding plate properties related to a transpressional zone near South America (figure 1) [52] impeding channelized magma flow through the crust.

## Data Availability

All presented new data are available in the manuscript and online supplementary material. Included data from previous studies are available from published sources highlighted in the manuscript and the GEOROC database (https://georoc.eu/). Electronic supplementary material is available online at [128].
